# Supplementary material for: Substance use disorders in refugee and migrant groups in Sweden: A nationwide cohort study of 1.2 million people
Source: PLoS Med. 2019 Nov 5;16(11):e1002944. doi: 10.1371/journal.pmed.1002944 (PMC6830745; doi:10.1371/journal.pmed.1002944)
Supplement: S1 STROBE Checklist — (DOCX) [file pmed.1002944.s001.docx]

**S1 STROBE Statement—checklist of items that should be included in reports of observational studies**

|  | Item No | Recommendation | Section, Paragraph number |
| --- | --- | --- | --- |
| **Title and abstract** | 1 | (*a*) Indicate the study’s design with a commonly used term in the title or the abstract  Substance use disorders in refugee and migrant groups in Sweden: a nationwide cohort study of 1.2 million people | Title & Abstract (methods & findings) |
|  |  | (*b*) Provide in the abstract an informative and balanced summary of what was done and what was found  Methods and findings: Using linked Swedish register data, we followed a cohort born between 1984 and 1997, from their 14th birthday or arrival in Sweden if later, until an International Classification of Diseases, 10th revision (ICD-10) diagnosis of substance use disorder (codes F10.X-19.X), emigration, death, or end of follow-up (31 December 2016). Refugee and non-refugee migrants were restricted to those from regions with at least 1000 refugees in the Swedish registers. We used Cox proportional hazards regression to estimate unadjusted and adjusted hazard ratios (HR) and 95% Confidence Intervals (95%CI) in refugee and non-refugee migrants, compared with Swedish-born individuals, for all substance use disorders (F10.X-19.X), alcohol use disorders (F10.X), cannabis use disorders (F12.X), and polydrug use disorders (F19.X). In adjusted analyses, we controlled for age, sex, birth year family income, family employment status, population density, and PTSD diagnosis. Our sample of 1,241,901 participants included 17,783 (1.4%) refugee and 104,250 (8.4%) non-refugee migrants, who predominantly originated from the Middle East and North Africa (41.4% and 33.7%, respectively), and were more economically disadvantaged at cohort entry (p<0.001) than the Swedish-born population. Refugee (adjusted HR: 0.52; 95%CI: 0.46-0.60) and non-refugee (aHR: 0.46; 95%CI: 0.43-0.49) migrants had similarly lower rates of all substance use disorders compared with Swedish-born individuals (crude incidence: 290.2 cases per 100,000 person-years; 95%CI: 287.3-293.1). Rates of substance use disorders in migrants converged to the Swedish-born rate over time, indicated by both earlier age-at-migration and longer time in Sweden. We observed similar patterns for alcohol, cannabis and polydrug use disorders, and for migrants from most regions-of-origin. Finally, while a PTSD diagnosis was over 5 times more common in refugees than the Swedish-born population, it had more pronounced effects on increased rates of substance use disorders in the Swedish-born population (aHR: 7.36, 95%CI: 6.79-7.96) than non-refugee migrants (HR: 4.88; 95%CI: 3.71-6.41; likelihood ratio test: p=0.01). Our findings highlight differences in the diagnosed incidence of substance use disorders detected in secondary care in Sweden, as recorded in the Swedish National Patient Register, but may have led to non-differential or differential under-ascertainment (by migrant status) of those only seen via primary care. | Abstract (Methods and findings) |
| Introduction | | |  |
| Background/rationale | 2 | Explain the scientific background and rationale for the investigation being reported  The epidemiological evidence for risk of substance use disorders in refugee and non-refugee migrant groups is still poorly understood… Many studies have been based on cross-sectional surveys [22,24–27], prevalence data [20,22,24,26,27], small samples [24,26,27], imprecise definitions of refugee status [18] or substance use behaviours rather than disorder [20,24,25], making it difficult to draw firm conclusions about the risk of substance use disorders in refugee and non-refugee migrant groups. | Introduction, paragraphs 2-4 |
| Objectives | 3 | State specific objectives, including any prespecified hypotheses  We therefore investigated whether the incidence of substance use disorders in refugees and non-refugee migrants from regions with substantial refugee flows to Sweden differed to the native-born population in a large, nationwide Swedish cohort, using high-quality linked register data. Specifically, we tested whether rates of substance use disorders would  •be were lower in refugee and non-refugee migrant populations in Sweden  •vary by region of origin,  •converge to the Swedish-born rate with earlier age-at-migration, and longer time lived in Sweden amongst migrant populations  •be independently associated with PTSD, that PTSD would be more common in refugee and non-refugee migrants (i.e. greater impact), but that PTSD would not modify the association between migrant status and substance use disorders | Introduction, paragraph 5 |
| Methods | | |  |
| Study design | 4 | Present key elements of study design early in the paper  i.e.: Methods paragraph 2  Using longitudinal Swedish register data from Psychiatry Sweden, a database of linked registers to explore the causes and outcomes of mental health disorders, we established an initial cohort of 1,345,320 people born between 1984 and 1997, of refugees, non-refugee migrants, and Swedish-born participants. We restricted the cohort to this birth period to ensure we could follow all participants diagnosed with an International Classification of Diseases, 10th revision, (ICD-10) substance use disorder (F10-19), which was introduced in Sweden in 1997. | Methods, paragraphs 2-7 |
| Setting | 5 | Describe the setting, locations, and relevant dates, including periods of recruitment, exposure, follow-up, and data collection  See above. | Methods, paragraphs, 2-5 |
| Participants | 6 | (*a*) *Cohort study*—Give the eligibility criteria, and the sources and methods of selection of participants. Describe methods of follow-up  See above. Also:  We only included refugee and non-refugee migrants who arrived in Sweden from 1 January 1998 (when refugee status was first recorded in the Immigration/Emigration Register (STATIV)) from regions-of-origin where data were available in the Swedish registers on at least 1,000 refugees, to permit valid comparisons between these two groups, consistent with our previous methodology [7]. Migrants from other regions (e.g. Western Europe, the Americas or Oceania) were excluded, as were children of migrants (second-generation migrants), defined as those born in Sweden to at least one foreign-born parent. Those not officially granted residence in Sweden (i.e. asylum seekers and undocumented migrants), and those diagnosed with non-affective psychosis (ICD-10 F20-29) or a substance use disorder (F10-19) before their 14th birthday were excluded. | Methods, paragraphs, 2-5 |
|  |  | (*b*) *Cohort study*—For matched studies, give matching criteria and number of exposed and unexposed | N/A |
| Variables | 7 | Clearly define all outcomes, exposures, predictors, potential confounders, and effect modifiers. Give diagnostic criteria, if applicable  Our primary outcome of interest was a first ICD-10 diagnosis of mental and behavioural disorders due to psychoactive substance use (F10.X-F19.X), as recorded in the National Patient Register…  Our primary exposure was migrant status, categorized as: refugee, non-refugee migrant, or Swedish-born using information from the Total Population register, STATIV, and the Multi-generational register….  Sex, birth year, family income, family employment status, population density and PTSD diagnosis were included as confounders. Using Lexis expansion, we also treated age as a time-varying covariate… | Methods, paragraphs 3-5 |
| Data sources/ measurement | 8* | For each variable of interest, give sources of data and details of methods of assessment (measurement). Describe comparability of assessment methods if there is more than one group  See above, and:  Family income was generated from the Longitudinal Integration Database for Health Insurance and Labour Market Statistics (LISA).  Similarly, we considered parental employment status (employed vs. unemployed), also retrieved from the LISA register. For migrants arriving after age 16 without parents, individual employment status was used. Population density was estimated for participants according to the “Small Area Marketing Statistic” neighbourhood (N=9,200) in which they were registered in their year of cohort entry according to the Total Population register. | Methods, paragraphs 2-5 |
| Bias | 9 | Describe any efforts to address potential sources of bias  Methods, paragraph 3:  In- and out-patient coverage are known to be complete after 1987 and 2001, respectively [32], for publicly-funded healthcare settings and privately-funded in-patient care, and around 80% complete for privately-funded outpatient settings.  Methods, paragraph 6:  To investigate possible biases introduced by excluding participants with missing covariate data, we re-ran our main unadjusted and adjusted Cox proportional hazards models for all substance use disorders, including people with missing income data (N=99,631) in a sensitivity analysis; when controlling for income, we included those missing income as a separate category on this covariate. | Methods, paragraphs 3,6.  Discussion, paragraphs 3, 4 |
| Study size | 10 | Explain how the study size was arrived at  Using longitudinal Swedish register data from Psychiatry Sweden, a database of linked registers to explore the causes and outcomes of mental health disorders, we established an initial cohort of 1,345,320 people born between 1984 and 1997, of refugees, non-refugee migrants, and Swedish-born participants. | Methods, paragraph 2 |
| Quantitative variables | 11 | Explain how quantitative variables were handled in the analyses. If applicable, describe which groupings were chosen and why  i.e. Methods, paragraph 3  Age-at-migration was categorised as Swedish-born, 0-6 years, 7-15 years, 16-19 years, 20+ years, consistent with age periods at which people in Sweden typically transition through the education system. Time in Sweden was categorized as Swedish-born, 0-4 years, 5-9 years, and 10+ years.  i.e. Methods, paragraph 4  We created five age categories: 14-17, 18-21, 22-25, 26-29, and 30-32.  i.e. Methods, paragraph 5  We then fitted Cox proportional hazards models for each outcome by migrant status and region-of-origin, estimating unadjusted and adjusted hazard ratios (HR) and 95% confidence intervals (95%CI) for these analyses. | Methods, paragraphs 3-5 |
| Statistical methods | 12 | (*a*) Describe all statistical methods, including those used to control for confounding  See (11). | Methods, paragraph 5 |
|  |  | (*b*) Describe any methods used to examine subgroups and interactions  We tested whether the effect of migrant status on substance use disorders was modified by PTSD, assessed formally via likelihood ratio tests [LRT]. | Methods, paragraph 5 |
|  |  | (*c*) Explain how missing data were addressed  We excluded participants with missing covariate data and compared them with the complete case sample.  And:  To investigate possible biases introduced by excluding participants with missing covariate data, we re-ran our main unadjusted and adjusted Cox proportional hazards models for all substance use disorders, including people with missing income data (N=99,631) in a sensitivity analysis; when controlling for income, we included those missing income as a separate category on this covariate. | Methods, paragraph 5 |
|  |  | (*d*) *Cohort study*—If applicable, explain how loss to follow-up was addressed | N/A |
|  |  | (*e*) Describe any sensitivity analyses  See (12c). Also:  In a further sensitivity analysis restricted to migrant samples (i.e. excluding the Swedish-born reference category to reduce multicollinearity), we investigated whether migrant status, region of origin, age-at-migration and time in Sweden had independent effects on risk of any substance use disorders. | Methods, paragraph 5 |

Continued on next page

| Results | | |  |
| --- | --- | --- | --- |
| Participants | 13* | (a) Report numbers of individuals at each stage of study—eg numbers potentially eligible, examined for eligibility, confirmed eligible, included in the study, completing follow-up, and analysed  From 1,345,320 participants, we excluded 103,419 people (7.7%) missing covariate data (S1 Figure), including family income (n=99,631; 7.4%), employment status (n=3,710; 0.3%) or address information in year of cohort entry (n=78; 0.01%)…  Our final analytical sample included 1,241,901 people living in Sweden | Results, paragraph 1, Figure 1 |
|  |  | (b) Give reasons for non-participation at each stage  See above |  |
|  |  | (c) Consider use of a flow diagram | Figure 1 |
| Descriptive data | 14* | (a) Give characteristics of study participants (eg demographic, clinical, social) and information on exposures and potential confounders  Our final analytical sample included 1,241,901 people living in Sweden (92.3% of the total sample), including 17,783 refugees (1.4%), 104,250 non-refugee migrants (8.4%), and 1,119,868 Swedish-born individuals (90.2%) (Table 1). The largest proportion of refugee (41.4%) and non-refugee migrants (33.7%) were from the Middle East & North Africa, (33.7%), while the smallest proportion of refugee (6.0%) and non-refugee migrants (11.8%) were from Eastern Europe & Russia and sub-Saharan Africa. | Results, paragraphs 2-3, Table 1 |
|  |  | (b) Indicate number of participants with missing data for each variable of interest  Participants with missing data were more likely to be refugee or non-refugee migrants, younger, male, from lower family income quintiles, unemployed families and more urban areas than participants in the final analytical cohort (all p<0.001; S2 Table). They were less likely to have received a diagnosis of any substance use disorder or PTSD (all p<0.001; S2 Table). | Results paragraph 1, S2 Table |
|  |  | (c) *Cohort study*—Summarise follow-up time (eg, average and total amount)  We identified 40,417 (2.9%) individuals who were first diagnosed with a substance use disorder in the inpatient or outpatient register over 14,439,302 person-years of follow-up (Table 1). | Results paragraph 3, Table 1 |
| Outcome data | 15* | *Cohort study*—Report numbers of outcome events or summary measures over time  See (14c) | Results paragraph 3, Table 1 |
| Main results | 16 | (*a*) Give unadjusted estimates and, if applicable, confounder-adjusted estimates and their precision (eg, 95% confidence interval). Make clear which confounders were adjusted for and why they were included  i.e. Results, paragraph 4  In an unadjusted model, both refugee (HR: 0.42; 95%CI: 0.37-0.48; p<0.001) and non-refugee (HR: 0.42; 95%CI: 0.39-0.44; p<0.001) migrants were less likely to have received a diagnosis for a substance use disorder than the Swedish-born population (Table 2), patterns which persisted following adjustment for age, sex, birth year, region, family income, family employment, population density, and PTSD diagnosis (HR_refugees_: 0.52; 95%CI: 0.46-0.60; p<0.001; HR_migrants_: 0.46; 95%CI: 0.43-0.49; p<0.001). | Results paragraphs 4-9, Tables 2-4, S3, S4, S6, S7 tables |
|  |  | (*b*) Report category boundaries when continuous variables were categorized  i.e. P8:  We generated four categories of population density: <25 people/km2, 25.1-250 people/km2, 250.1-2,500 people/km2, and >2,500 people/km2 to control for urban effects. | Methods, paragraph 5 |
|  |  | (*c*) If relevant, consider translating estimates of relative risk into absolute risk for a meaningful time period  i.e. Results, paragraph 4  The crude incidence of substance use disorders was higher in the Swedish-born population (290.2 per 100,000 person-years; 95%CI: 287.3-293.1) than non-refugee migrants (141.8 per 100,000 person-years; 95%CI 134.0-150.0) and refugees (149.3 per 100,000 person-years, 95%CI: 130.9-170.2). | Results, paragraph 4 |
| Other analyses | 17 | Report other analyses done—eg analyses of subgroups and interactions, and sensitivity analyses  i.e. Results, paragraph 5  In sensitivity analyses in a larger cohort of 1.34m participants, including 99,631 missing data on income, there was little evidence that excluding this group biased our findings (S3 Table).  i.e. Results, paragraph 6  For all outcomes, we found dose-response relationships between rates of substance use disorders and earlier age-at-migration or longer time in Sweden (Table 4 and S4 Table), such that hazard ratios tended to converge to the Swedish-born rate over time. | Results, paragraph 5-9, Tables 3-5, S2-S8 tables |
| Discussion | | |  |
| Key results | 18 | Summarise key results with reference to study objectives  In our large, nationwide cohort, refugee and non-refugee migrants were substantially less likely to be diagnosed with a substance use disorder than Swedish-born individuals, extending to alcohol, cannabis, and polydrug disorders independently. We found no evidence of differences in incidence rates between these two migrant groups, with the possible exception of polydrug use disorders which may have been elevated amongst refugees compared with non-refugee migrants. Rates were lower for migrants from all regions-of-origin, with the exception of raised rates of cannabis use disorders for migrants from sub-Saharan Africa relative to the Swedish-born population. Rates in migrants converged to the Swedish-born rate over time, with dose-response patterns for both age-at-migration and time in Sweden. Finally, we found strong evidence that individuals with PTSD were more likely to be diagnosed with a substance use disorder than those without a PTSD diagnosis, a relative effect more pronounced amongst Swedish-born participants in contrast to our hypothesis. Nonetheless, PTSD may have had greater impact on the incidence of substance abuse disorders in migrant groups given its higher occurrence than in the Swedish-born population. | Discussion, paragraph 1 |
| Limitations | 19 | Discuss limitations of the study, taking into account sources of potential bias or imprecision. Discuss both direction and magnitude of any potential bias  i.e. Our longitudinal design in a comprehensive nationwide sample allowed us to obtain precise estimates of the incidence of substance use disorders in refugees and non-refugee migrants in comparison to the Swedish-born population for the first time….  And:  There were also notable limitations of our study. Most crucially, our incidence results were based on substance use disorders diagnosed in secondary care, including emergency department visits, but excluding contacts only seen within primary care. Here, non-differential case ascertainment would have led us to under-estimate the incidence of substance use disorders in Sweden, particularly where  And:  Our results should also be interpreted in the context of the validity of diagnoses made in clinical practice. In general, psychiatric diagnoses in the National Patient Register are valid [35], though direct evidence for substance use disorders remains to be established. Nonetheless, diagnoses in our study were based on those made following inpatient admission or specialised outpatient treatment, and we have no reason to believe they would not be valid. There is direct evidence that Swedish register diagnoses of PTSD have good validity [44]. | Discussion, paragraphs 2-5 |
| Interpretation | 20 | Give a cautious overall interpretation of results considering objectives, limitations, multiplicity of analyses, results from similar studies, and other relevant evidence  i.e.  Lower rates of substance use disorders – which we observed to a similar extent in both refugee and non-refugee migrant groups from the same regions of origin – may be attributable to various factors, including the ‘healthy immigrant effect’ or sociocultural and religious differences in attitudes and behaviours towards substance use. | Discussion, paragraphs 7-9 |
| Generalisability | 21 | Discuss the generalisability (external validity) of the study results  If generalisable, such high levels of psychiatric morbidity, and potential convergence of rates in migrant groups over time to those in the background population, will present a fundamental public health concern for many nations across the globe. | Discussion, paragraph 9 |
| Other information | | |  |
| Funding | 22 | Give the source of funding and the role of the funders for the present study and, if applicable, for the original study on which the present article is based  See Financial disclosure statement. | Financial disclosure statement |

*Give information separately for cases and controls in case-control studies and, if applicable, for exposed and unexposed groups in cohort and cross-sectional studies.

**Note:** An Explanation and Elaboration article discusses each checklist item and gives methodological background and published examples of transparent reporting. The STROBE checklist is best used in conjunction with this article (freely available on the Web sites of PLoS Medicine at http://www.plosmedicine.org/, Annals of Internal Medicine at http://www.annals.org/, and Epidemiology at http://www.epidem.com/). Information on the STROBE Initiative is available at www.strobe-statement.org.
