# Supplementary material for: Substance use disorders in refugee and migrant groups in Sweden: A nationwide cohort study of 1.2 million people
Source: PLoS Med. 2019 Nov 5;16(11):e1002944. doi: 10.1371/journal.pmed.1002944 (PMC6830745; doi:10.1371/journal.pmed.1002944)
Supplement: S1 Text — (DOCX) [file pmed.1002944.s002.docx]

**S1 Text: Original protocol**

The original protocol is available at protocols.io and reproduced here. It was originally developed for the M.Sc. dissertation submitted by Samantha Harris as part of her degree requirements for an M.Sc. in Clinical Mental Health Sciences at UCL, London, UK

**Name**: Samantha Harris

**Supervisor**: Dr. James Kirkbride

**Provisional project title**:

Admission for substance-induced disorders in refugee and migrant groups in Sweden

**Background**

Refugees have reported living in extreme conditions of poverty, and the negative impact this has on their psychological well-being (Freedom from Torture report, 2013). Previous literature suggests that refugees are at a higher risk of developing psychosis as opposed to natives as well as non-refugee migrants (Hollander et al., 2016). We do not yet have a clear idea of their risk of substance abuse, although it has been shown that this is a risk people face who have suffered traumatic experiences (Stewart, 1996). The objective of this study is to determine whether refugees and non-refugee migrants are at an elevated risk of substance misuse relative to the Swedish-born population. We will consider the impact of PTSD on the association between refugee status and substance use disorders.

**Method**

**Setting**

Sweden is said to have taken in more refugees than any other European country, making it an ideal country to conduct this research on. The study will follow similar methods as Hollander and colleagues, who conducted their study using the Psychiatry Sweden database, a large database of linked national registers that provides a variety of information on everyone living in Sweden, such as date of birth, sex and country of birth.

**Measures**

Using the information from the database, it will be possible to calculate hazard ratios for substance use disorders admissions in refugees, non-refugee migrants, and Swedish natives. We will also consider alcohol use, cannabis use and polydrug use disorders separately.

**Sample**

Participants will include Swedish natives, born to two Swedish parents, non-refugee migrants, and refugees living in Sweden. The cohort will comprise of all people born between 1984-1997, living in Sweden on or after their 14^th^ birthday and followed until 31 December 2016, substance use disorder, emigration or death, whichever was sooner. We will restrict the cohort to refugees and non-refugee migrants from regions-of-origin where at least 1000 refugees are recorded in the Swedish total population register to permit meaningful comparisons.

**Study procedures**

The study will establish a prospective cohort from the database. The main exposure of interest examined will be refugee status, while the main outcome is substance misuse related admissions. People with ICD-10 substance use disorders (F10-19) will be identified from the National Patient Register. PTSD will be recorded in the NPR according to ICD-10 43.1. Possible confounders will be considered, such as: age at arrival, age and sex, disposable income employment status and population density, as these may all be related to substance misuse.

**Approach to analysis**

We will gather basic descriptive statistics, and crude incidence rates for each of the three groups. Similarly to Hollander and colleagues, we will fit Cox proportional hazards models in order to calculate hazard ratios and confidence intervals. PTSD will be considered as a confounder and effect modifier of the refugee-substance use disorder association.

**References:**

- - Hollander, A.-C., Dal, H., Lewis, G., Magnusson, C., Kirkbride, J. B., & Dalman, C. (2016). Refugee migration and risk of schizophrenia and other non-affective psychoses: cohort study of 1.3 million people in Sweden. *Bmj-British Medical Journal, 352*. doi:10.1136/bmj.i1030
  - Stewart, S. H. (1996). Alcohol abuse in individuals exposed to trauma: A critical review. *Psychological Bulletin, 120*(1), 83-112. doi:10.1037//0033-2909.120.1.83
  - Freedom from Torture report, Jo Pettitt, 2013
