# Supplementary figures and images for: Substance use disorders in refugee and migrant groups in Sweden: A nationwide cohort study of 1.2 million people
Source: PLoS Med. 2019 Nov 5;16(11):e1002944. doi: 10.1371/journal.pmed.1002944 (PMC6830745; doi:10.1371/journal.pmed.1002944)

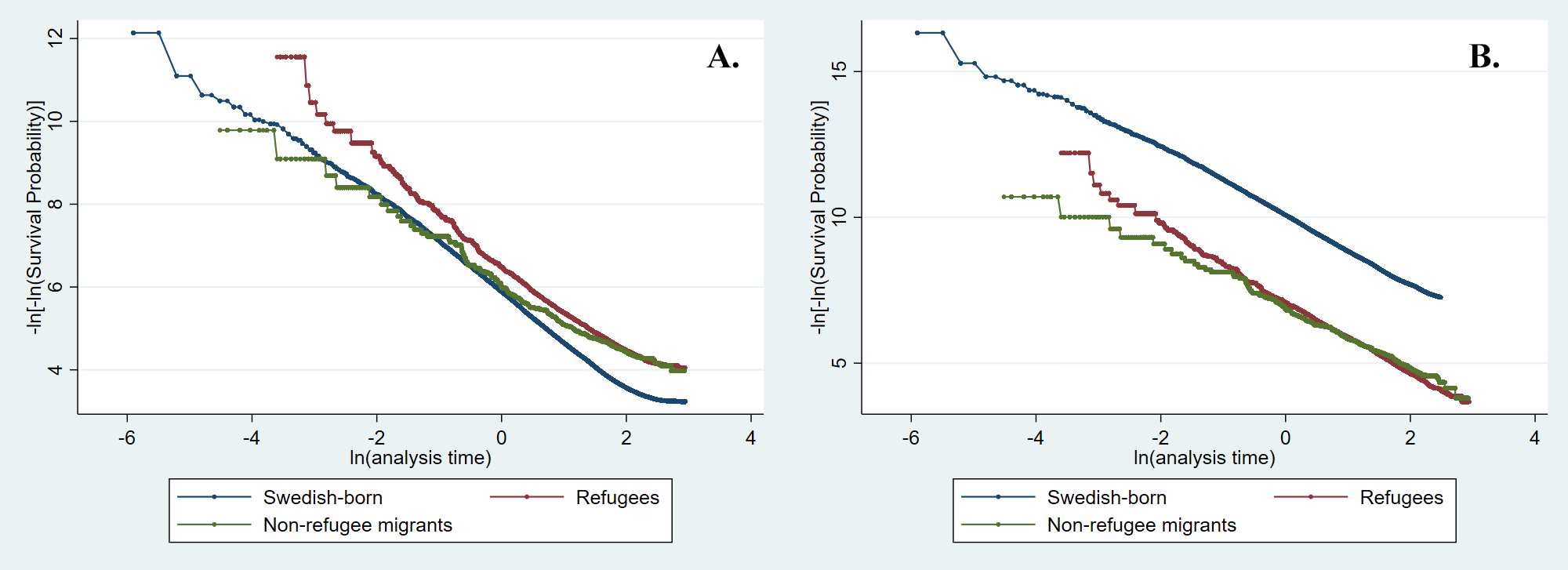

Supplement: S1 Fig — (A) Crude model and (B) adjusted model of log-log plots of Schoenfeld residuals by log time, following Cox proportional hazards modelling of any substance use disorder. Both panels indicate substantial departure from the proportional hazards assumptions for the non-refugee migrant group (green line), although a less severe violation of this assumption for refugee migrants (red line) relative to the Swedish-born population (blue line). See S5 Table for possible effect of bias on estimates. (TIF) [file pmed.1002944.s003.tif]
