# Supplementary material for: Substance use disorders in refugee and migrant groups in Sweden: A nationwide cohort study of 1.2 million people
Source: PLoS Med. 2019 Nov 5;16(11):e1002944. doi: 10.1371/journal.pmed.1002944 (PMC6830745; doi:10.1371/journal.pmed.1002944)
Supplement: S1 Table — (DOCX) [file pmed.1002944.s004.docx]

**S1 Table: Region-of-origin classification and basic sample characteristics of migrant groups**

| **Region of origin^1^** | **SMA classification^2^** | **Cohort characteristics^3^** | | **Countries^4^** |
| --- | --- | --- | --- | --- |
|  |  | Cases (%) | PYAR (%) |  |
| Sub-Saharan Africa | West Africa | 59 (4.1) | 17,577 (1.7) | Benin, Burkina Faso, Cape Verde, Gambia, Ghana, Guinea, Guinea Bissau, Ivory Coast, Liberia, Mali, Mauritania, Niger, Nigeria, Senegal, Sierra Leone, Togo |
|  | East Africa | 137 (9.5) | 103,944 (10.3) | Djibouti, Eritrea, Ethiopia ,Somalia |
|  | Africa, other | 61 (4.2) | 29,656 (2.9) | Angola, Botswana, Burundi, Cameroon, Central African Republic, Chad, Comoros, Congo, Democratic Republic of Congo, Equatorial Guinea, Gabon, Kenya, Lesotho, Madagascar, Malawi, Mauritius, Mozambique, Namibia, Rwanda, Sao Tome and Principe, Seychelles, South Africa, Swaziland, Tanzania, Uganda, Zambia, Zanzibar |
| Asia | Central Asia | 146 (10.1) | 109,511 (10.8) | Afghanistan, Armenia, Azerbaijan, Bangladesh, Bhutan, Georgia, India, Kazakhstan, Kyrgyzstan, Maldives, Nepal, Pakistan, Sri Lanka, Tajikistan, Turkmenistan |
|  | Northeast Asia | 30 (2.1) | 44,936 (4.4) | China, Japan, Mongolia, People’s Republic of Korea, South Korea, Taiwan |
|  | Southeast Asia | 90 (6.2) | 69,395 (6.9) | Brunei, Cambodia, East Timor, Hong Kong, Indonesia, Laos, Malaysia, Myanmar, Philippines, Singapore, Thailand, Vietnam |
| Eastern Europe & Russia | Eastern Europe | 159 (11.0) | 102,520 (10.1) | Albania, Belarus, Bulgaria, Czech Republic, Hungary, Moldova, Poland, Romania, Slovakia, Ukraine |
|  | Former Yugoslavia | 125 (8.6) | 102,886 (10.2) | Bosnia Herzegovina, Croatia, Kosovo, Macedonia, Montenegro, Serbia, Slovenia |
|  | Russia & the Baltic States | 107 (7.4) | 52,197 (5.2) | Estonia, Latvia, Lithuania, Russia |
| Middle East & North Africa | Iran | 90 (6.2) | 36,789 (3.6) | Iran |
|  | Iraq | 324 (22.4) | 248,093 (24.5) | Iraq |
|  | Middle East, other | 87 (6.0) | 80,961 (8.0) | Bahrain, Cyprus, Israel, Jordan, Kuwait, Lebanon, Oman, Palestine, Qatar, Saudi Arabia, Syria, United Arab Emirates, Yemen, Turkey |
|  | North Africa | 31 (2.1) | 13,499 (1.3) | Algeria, Egypt, Libya, Morocco, Tunisia |

SMA: Swedish Migration Agency; PYAR: Person-years at-risk

^1^Predefined by authors for analysis, based on SMA classification. Sweden not shown.

^2^Categories provided by the Swedish Migration Agency for research purposes, reflecting major migrant and refugee flows to Sweden

^3^Cases of any substance induced disorder

^4^Countries of origin, as defined by the Swedish Migration Agency, in each SMA category. Country-level data is not made available by the SMA/Statistics Sweden for research purposes.
