# Supplementary material for: Substance use disorders in refugee and migrant groups in Sweden: A nationwide cohort study of 1.2 million people
Source: PLoS Med. 2019 Nov 5;16(11):e1002944. doi: 10.1371/journal.pmed.1002944 (PMC6830745; doi:10.1371/journal.pmed.1002944)
Supplement: S2 Table — (DOCX) [file pmed.1002944.s005.docx]

**S2 Table: Cohort characteristics by missingness status**

|  | **Analytic cohort** | | **Missing sample^1^** | | **χ^2^ p-value** |
| --- | --- | --- | --- | --- | --- |
|  | **Cases** | **%** | **Cases** | **%** |  |
| **Outcome** |  |  |  |  |  |
| Any substance use disorder | 40,417 | 3.25 | 382 | 0.37 | 2,702.3 (1); p<0.001 |
| Alcohol use disorder | 30,251 | 2.44 | 260 | 0.25 | 2,055.3 (1); p<0.001 |
| Cannabis use disorder | 4,734 | 0.38 | 36 | 0.03 | 324.2 (1); p<0.001 |
| Poly-drug use disorder | 7,852 | 0.63 | 17 | 0.02 | 622.6 (1); p<0.001 |
| **Migrant status** |  |  |  |  | 52,039 (2); p<0.001 |
| Swedish-born | 1,119,868 | 90.2 | 68,943 | 66.7 |  |
| Non-refugee migrants | 104,250 | 8.4 | 28,152 | 27.2 |  |
| Refugee migrants | 17,783 | 1.4 | 6,324 | 6.1 |  |
| **Sex** |  |  |  |  | 13.0 (1); p<0.001 |
| Female | 606,065 | 48.8 | 49,866 | 48.2 |  |
| Male | 635,836 | 51.2 | 53,553 | 51.8 |  |
| **Age (years) (median; IQR)** | 21.5 | 18.5-24.6 | 14.7 | 14.4-16.0 | Z=387.8; p<0.001^2^ |
| **Region** |  |  |  |  | 52,210 (4); p<0.001 |
| Sweden | 1,119,868 | 90.2 | 68,943 | 66.7 |  |
| Eastern Europe& Russia | 31,302 | 2.5 | 8,775 | 8.5 |  |
| Asia | 29,645 | 2.4 | 9,654 | 9.3 |  |
| Middle East & North Africa | 42,451 | 3.4 | 10,327 | 10.0 |  |
| Sub-Saharan Africa | 18,635 | 1.5 | 5,720 | 5.5 |  |
| **Family income** |  |  |  |  |  |
| Quintile 1 (Lowest) | 80,534 | 6.5 | 395 | 10.4 | 2634.4 (4); p<0.001 |
| Quintile 2 | 98,341 | 7.9 | 803 | 21.2 |  |
| Quintile 3 | 225,436 | 18.2 | 1,461 | 38.6 |  |
| Quintile 4 | 405,421 | 32.7 | 722 | 19.1 |  |
| Quintile 5 (Highest) | 432,169 | 34.8 | 407 | 10.7 |  |
| **Family employment** |  |  |  |  |  |
| Unemployed | 202,203 | 16.3 | 95,205 | 100.0 | 18,244.9 (1); p<0.001 |
| Employed | 1,039,698 | 83.7 | 8 | 0.0 |  |
| **Population density** |  |  |  |  | 2280.2 (3); p<0.001 |
| 0-25 pp/km^2^ | 296,434 | 23.9 | 21,399 | 20.9 |  |
| 25.1-250 pp/km^2^ | 673,477 | 54.2 | 52,233 | 50.9 |  |
| 250.1-2,500 pp/km^2^ | 194,846 | 15.7 | 20,502 | 20.0 |  |
| 2,500.1 or more pp/km^2^ | 77,144 | 6.2 | 8,489 | 8.3 |  |
| **PTSD diagnosis** |  |  |  |  |  |
| Diagnosed PTSD | 3,256 | 0.3 | 206 | 0.2 | 14.8 (1); p<0.001 |

IQR: interquartile range; PTSD: Post-traumatic stress disorder

^1^Distribution of those excluded from analytical cohort due to missing data (N=103,419) by available outcome, exposure and covariate data

^2^Mann-Whitney U-test
