# Supplementary material for: Substance use disorders in refugee and migrant groups in Sweden: A nationwide cohort study of 1.2 million people
Source: PLoS Med. 2019 Nov 5;16(11):e1002944. doi: 10.1371/journal.pmed.1002944 (PMC6830745; doi:10.1371/journal.pmed.1002944)
Supplement: S3 Table — HR, hazard ratio (DOCX) [file pmed.1002944.s006.docx]

**S3 Table: Unadjusted and adjusted hazard ratios for substance use disorders by migrant status in sensitivity analysis of enlarged sample (N=1,341,532) with adjustment for income^1^**

|  | **Cases** | **%** | **Unadjusted** | |  |  | **Adjusted** | | |  |
| --- | --- | --- | --- | --- | --- | --- | --- | --- | --- | --- |
|  |  |  | **HR** | **95%CI** | | **p-value** | **HR^2^** | **95%CI** | | **p-value** |
| **Substance use disorder** |  |  |  |  |  |  |  |  | |  |
| Swedish-born | 39,183 | 3.3 | 1 |  |  |  | 1 |  |  |  |
| Non-refugee migrants | 1,300 | 1.0 | 0.39 | 0.37 | 0.41 | <0.001 | 0.46 | 0.43 | 0.49 | <0.001 |
| Refugees | 243 | 1.0 | 0.38 | 0.34 | 0.44 | <0.001 | 0.52 | 0.46 | 0.60 | <0.001 |
| **Alcohol use disorder** |  |  |  |  |  |  |  |  |  |  |
| Swedish-born | 29,589 | 2.5 | 1 |  |  |  | 1 |  |  |  |
| Non-refugee migrants | 742 | 0.6 | 0.28 | 0.26 | 0.31 | <0.001 | 0.35 | 0.32 | 0.39 | <0.001 |
| Refugees | 131 | 0.6 | 0.26 | 0.22 | 0.31 | <0.001 | 0.38 | 0.31 | 0.46 | <0.001 |
| **Cannabis use disorder** |  |  |  |  |  |  |  |  |  |  |
| Swedish-born | 4,394 | 0.4 | 1 |  |  |  | 1 |  |  |  |
| Non-refugee migrants | 311 | 0.2 | 0.89 | 0.79 | 1.00 | 0.04 | 0.81 | 0.70 | 0.93 | <0.001 |
| Refugees | 54 | 0.2 | 0.81 | 0.62 | 1.07 | 0.14 | 0.96 | 0.72 | 1.29 | 0.78 |
| **Poly-drug use disorder** |  |  |  |  |  |  |  |  |  |  |
| Swedish-born | 7,558 | 0.6 | 1 |  |  |  | 1 |  |  |  |
| Non-refugee migrants | 252 | 0.2 | 0.45 | 0.40 | 0.51 | <0.001 | 0.44 | 0.37 | 0.51 | <0.001 |
| Refugees | 55 | 0.2 | 0.53 | 0.40 | 0.68 | <0.001 | 0.58 | 0.44 | 0.78 | <0.001 |

95%CI: 95% confidence interval

^1^Participants with missing income data were included in cohort with those originally missing family income modelled as a separate category when controlling for family income

^2^Adjusted for age, sex, birth year, family employment, population density, PTSD diagnosis and family income
