# Supplementary material for: Substance use disorders in refugee and migrant groups in Sweden: A nationwide cohort study of 1.2 million people
Source: PLoS Med. 2019 Nov 5;16(11):e1002944. doi: 10.1371/journal.pmed.1002944 (PMC6830745; doi:10.1371/journal.pmed.1002944)
Supplement: S4 Table — HR, hazard ratio (DOCX) [file pmed.1002944.s007.docx]

**S4 Table: Unadjusted and adjusted hazard ratios by age-at-migration and time in Sweden for specific substance use disorders**

|  | **N** | **%** | **Unadjusted** | | |  | **Adjusted** | | |  | |
| --- | --- | --- | --- | --- | --- | --- | --- | --- | --- | --- | --- |
| **Disorder** |  |  | **HR** | **95%CI** | | **p-value** | **HR^1^** | **95%CI** | | | **p-value** |
| **Alcohol use disorders** |  |  |  |  |  |  |  |  | | |  |
| Swedish-born | 29,444 | 2.6 | 1 |  |  |  | 1 |  |  | |  |
| *Age-at-migration* |  |  |  |  |  |  |  |  |  | |  |
| 0-6 years | 69 | 1.1 | 0.54 | 0.42 | 0.68 | <0.001 | 0.65 | 0.51 | 0.82 | | <0.001 |
| 7-15 years | 446 | 1.1 | 0.45 | 0.41 | 0.50 | <0.001 | 0.36 | 0.33 | 0.40 | | <0.001 |
| 16-19 years | 172 | 0.6 | 0.27 | 0.23 | 0.32 | <0.001 | 0.23 | 0.18 | 0.28 | | <0.001 |
| 20+ years | 120 | 0.3 | 0.13 | 0.11 | 0.15 | <0.001 | 0.26 | 0.18 | 0.37 | | <0.001 |
| *Time in Sweden* |  |  |  |  |  |  |  |  |  | |  |
| 0-4 years | 676 | 0.6 | 0.28 | 0.26 | 0.30 | <0.001 | 0.32 | 0.29 | 0.35 | | <0.001 |
| 5-9 years | 104 | 1.1 | 0.49 | 0.41 | 0.60 | <0.001 | 0.54 | 0.44 | 0.65 | | <0.001 |
| 10+ years | 27 | 1.6 | 0.83 | 0.57 | 1.22 | 0.34 | 0.96 | 0.66 | 1.40 | | 0.82 |
|  |  |  |  |  |  |  |  |  |  | |  |
| **Cannabis use disorders** |  |  |  |  |  |  |  |  |  | |  |
| Swedish-born | 4,381 | 0.4 | 1 |  |  |  | 1 |  |  | |  |
| *Age-at-migration* |  |  |  |  |  |  |  |  |  | |  |
| 0-6 years | 23 | 0.4 | 1.33 | 0.88 | 2.00 | 0.17 | 0.94 | 0.62 | 1.42 | | 0.77 |
| 7-15 years | 234 | 0.6 | 1.68 | 1.47 | 1.92 | <0.001 | 0.85 | 0.74 | 0.98 | | 0.03 |
| 16-19 years | 82 | 0.3 | 0.93 | 0.75 | 1.16 | 0.51 | 0.47 | 0.34 | 0.66 | | <0.001 |
| 20+ years | 14 | 0.0 | 0.11 | 0.07 | 0.19 | <0.001 | 0.12 | 0.06 | 0.26 | | <0.001 |
| *Time in Sweden* |  |  |  |  |  |  |  |  |  | |  |
| 0-4 years | 303 | 0.3 | 0.90 | 0.80 | 1.01 | 0.08 | 0.79 | 0.68 | 0.92 | | 0.002 |
| 5-9 years | 41 | 0.4 | 1.41 | 1.04 | 1.92 | 0.03 | 0.92 | 0.67 | 1.25 | | 0.59 |
| 10+ years | 9 | 0.5 | 2.11 | 1.10 | 4.06 | 0.03 | 1.53 | 0.79 | 2.95 | | 0.20 |
|  |  |  |  |  |  |  |  |  |  | |  |
| **Poly-drug use disorders** |  |  |  |  |  |  |  |  |  | |  |
| Swedish-born | 7,553 | 0.7 | 1 |  |  |  | 1 |  |  | |  |
| *Age-at-migration* |  |  |  |  |  |  |  |  |  | |  |
| 0-6 years | 20 | 0.3 | 0.74 | 0.47 | 1.14 | 0.17 | 0.92 | 0.59 | 1.44 | | 0.73 |
| 7-15 years | 174 | 0.4 | 0.74 | 0.64 | 0.87 | <0.001 | 0.47 | 0.40 | 0.55 | | <0.001 |
| 16-19 years | 75 | 0.3 | 0.51 | 0.41 | 0.64 | <0.001 | 0.24 | 0.17 | 0.33 | | <0.001 |
| 20+ years | 30 | 0.1 | 0.16 | 0.11 | 0.22 | <0.001 | 0.13 | 0.07 | 0.24 | | <0.001 |
| *Time in Sweden* |  |  |  |  |  |  |  |  |  | |  |
| 0-4 years | 254 | 0.2 | 0.47 | 0.41 | 0.53 | <0.001 | 0.39 | 0.33 | 0.46 | | <0.001 |
| 5-9 years | 37 | 0.4 | 0.79 | 0.57 | 1.09 | 0.15 | 0.92 | 0.67 | 1.28 | | 0.62 |
| 10+ years | 8 | 0.5 | 1.22 | 0.61 | 2.44 | 0.57 | 1.26 | 0.63 | 2.52 | | 0.52 |

HR: Hazard ratio; 95%CI: 95% confidence interval

^1^Adjusted for age, sex, birth year, family income, family employment, population density, PTSD diagnosis
