# Supplementary material for: Substance use disorders in refugee and migrant groups in Sweden: A nationwide cohort study of 1.2 million people
Source: PLoS Med. 2019 Nov 5;16(11):e1002944. doi: 10.1371/journal.pmed.1002944 (PMC6830745; doi:10.1371/journal.pmed.1002944)
Supplement: S5 Table — AIC, Akaike’s Information Criterion (DOCX) [file pmed.1002944.s008.docx]

**S5 Table: Comparison of Akaike’s Information Criterion (AIC) scores from adjusted Cox proportional hazards regression for main exposures on substance use disorder outcomes^1^**

|  | **AIC for each outcome** | | | |
| --- | --- | --- | --- | --- |
| **Exposures** | **Any** | **Alcohol** | **Cannabis** | **Poly-drug** |
| Migrant status | 1,111,183 | 833,037 | 127,944 | 212,690 |
| Region-of-origin | 1,111,171 | 833,026 | **127,919** | **212,660** |
| Age-at-migration | **1,111,123** | **832,999** | 127,920 | 212,668 |
| Time in Sweden | 1,111,140 | **832,999** | 127,944 | 212,671 |

^1^Lower AIC scores denote better model fit within each outcome. AIC scores are not comparable between outcomes. We could not model all exposures together in a joint model due to multicollinearity (same reference category: Swedish-born). AICs are reported for each exposure in a multivariable Cox proportional hazards model, adjusted for age, sex, birth year, family income, family employment, population density and PTSD diagnosis. Lowest AIC scores for each outcome shown in **bold**
