# Supplementary material for: Substance use disorders in refugee and migrant groups in Sweden: A nationwide cohort study of 1.2 million people
Source: PLoS Med. 2019 Nov 5;16(11):e1002944. doi: 10.1371/journal.pmed.1002944 (PMC6830745; doi:10.1371/journal.pmed.1002944)
Supplement: S6 Table — (DOCX) [file pmed.1002944.s009.docx]

**S6 Table: Risk of any substance use disorder in migrant sub-sample after mutual adjustment for all exposures and confounders^1^**

| **Variable** | **Adjusted HR^2^** | **95%CI** | | **p-value** |
| --- | --- | --- | --- | --- |
| **Migrant status** |  |  | |  |
| Non-refugee migrants | 1 |  |  |  |
| Refugees | 1.14 | 0.98 | 1.32 | 0.10 |
|  |  |  |  |  |
| **Region-of-origin** |  |  |  |  |
| Eastern Europe & Russia | 1 |  |  |  |
| Asia | 0.82 | 0.70 | 0.95 | 0.01 |
| Middle East & North Africa | 0.81 | 0.70 | 0.92 | 0.002 |
| Sub-Saharan Africa | 1.11 | 0.95 | 1.31 | 0.19 |
|  |  |  |  |  |
| **Age-at-migration** |  |  |  |  |
| 0-6 years | 1 |  |  |  |
| 7-15 years | 1.14 | 0.83 | 1.56 | 0.42 |
| 16-19 years | 0.77 | 0.52 | 1.09 | 0.14 |
| 20+ years | 0.56 | 0.35 | 0.90 | 0.02 |
|  |  |  |  |  |
| **Time in Sweden** |  |  |  |  |
| 0-4 years | 1 |  |  |  |
| 5-9 years | 1.42 | 1.15 | 1.76 | 0.001 |
| 10+ years | 2.41 | 1.54 | 3.77 | <0.001 |

HR: Hazard ratio; 95%CI: 95% confidence interval; AIC: Akaike’s information criterion

^1^Restricted to migrants and refugees, excluding the Swedish-born population to minimise issue of multicollinearity between exposures (all originally categorized with Swedish-born population as reference category). New reference categories are non-refugee migrants (migrant status), Eastern Europe & Russia (region-of-origin), 0-6 years (age-at-migration) and 0-4 years (time in Sweden)

^2^Mutual adjustment for migrant status, region-of-origin, age-at-migration, time in Sweden, age, sex, birth year, family income, family employment, population density, PTSD diagnosis
