# Supplementary material for: Substance use disorders in refugee and migrant groups in Sweden: A nationwide cohort study of 1.2 million people
Source: PLoS Med. 2019 Nov 5;16(11):e1002944. doi: 10.1371/journal.pmed.1002944 (PMC6830745; doi:10.1371/journal.pmed.1002944)
Supplement: S7 Table — (DOCX) [file pmed.1002944.s010.docx]

**S7 Table: Examining evidence for violation of proportional hazards by migrant status for all substance use disorders**

| **Follow-up period^1^** | **Cases** | **(%)^2^** | **Adjusted HR^3^** | **95% CI** | |
| --- | --- | --- | --- | --- | --- |
| **Period 1 (0-3.16 years of follow-up)** |  |  |  |  |  |
| Swedish-born | 12,680 | (95.1) | 1 |  |  |
| Non-refugee migrants | 534 | (4.0) | 0.41 | 0.36 | 0.46 |
| Refugees | 119 | (0.9) | 0.55 | 0.45 | 0.67 |
| Total cases | 13,333 | (33.0) | - | - | - |
| **Period 2 (3.16-5.92 years of follow-up)** |  |  |  |  |  |
| Swedish-born | 12,890 | (96.6) | 1 |  |  |
| Non-refugee migrants | 386 | (2.9) | 0.46 | 0.41 | 0.52 |
| Refugees | 60 | (0.4) | 0.42 | 0.32 | 0.55 |
| Total cases | 13,336 | (33.0) | - | - | -- |
| **Period 2 (5.92-18-96 years of follow-up)** |  |  |  |  |  |
| Swedish-born | 13,391 | (97.4) | 1 |  |  |
| Non-refugee migrants | 303 | (2.2) | 0.55 | 0.48 | 0.63 |
| Refugees | 44 | (0.3) | 0.61 | 0.45 | 0.84 |
| Total cases | 13,738 | (34.0) | - | - | - |

HR: Hazard ratio; 95%CI: 95% confidence interval.

^1^Time was split into 3 categories with equal numbers of cases in each follow-up period. There was evidence from proportional hazards test of departure from proportional hazards for non-refugee migrants (p<0.001) although not refugees (p=0.28) in our fully adjusted model (see Supplemental Figure 2). This suggested that at all three time periods rates were lower for refugee and non-refugee migrants than the Swedish-born population, though rates of substance use disorders in non-refugee migrant groups became higher over the follow-up period, consistent with models of longer time in Sweden (see Table 4, S4 and S6 Tables).

^2^Percentages for “Total Cases” relative to overall number of substance use disorders in whole cohort. Percentages for specific group are percentage of total cases in each period.

^3^Adjusted for age, sex, birth year, , family income, family employment, population density, PTSD diagnosis
