# Supplementary material for: Substance use disorders in refugee and migrant groups in Sweden: A nationwide cohort study of 1.2 million people
Source: PLoS Med. 2019 Nov 5;16(11):e1002944. doi: 10.1371/journal.pmed.1002944 (PMC6830745; doi:10.1371/journal.pmed.1002944)
Supplement: S8 Table — (DOCX) [file pmed.1002944.s011.docx]

**S8 Table: All-cause mortality rate by migrant status**

| **Migrant status** | **Deaths** | **%** | **Person-years** | **Rate ^1^** | **95% CI** | |
| --- | --- | --- | --- | --- | --- | --- |
| Swedish-born | 2,627 | 0.2 | 13,990,212 | 18.78 | 18.07 | 19.51 |
| Non-refugee migrants | 98 | 0.1 | 974,877 | 10.05 | 8.25 | 12.25 |
| Refugees | 19 | 0.1 | 157,071 | 12.10 | 7.72 | 18.96 |

**^1^** Per 100,000 person years

95%CI: 95% confidence interval
