# Supplementary material for: Substance use disorders in refugee and migrant groups in Sweden: A nationwide cohort study of 1.2 million people
Source: PLoS Med. 2019 Nov 5;16(11):e1002944. doi: 10.1371/journal.pmed.1002944 (PMC6830745; doi:10.1371/journal.pmed.1002944)
Supplement: Attachment — Submitted filename: Response to reviewers PLoS Medicine.docx [file pmed.1002944.s012.docx]

| **Dr James B. Kirkbride**  Reader in Epidemiology |  |
| --- | --- |
| **Division of Psychiatry** | |
| 8^th^ August 2019 |  |

**Dear Reviewers**

**Thank you for your most helpful reviews of our manuscript. Please see below for our point-by-point response to all editorial and reviewer comments.**

**We have tracked all changes in the revised manuscript and supplemental files. All page and line numbers in our response to reviewers are based on the tracked change version of the file. Our responses to the editorial and reviewer comments are in bold below. Quoted text from the manuscript is included in quotation marks with added sections of text underlined for clarity.**

**Yours faithfully**

**James Kirkbride**

CC: [rturner@plos.org](mailto:rturner@plos.org)

Dear Dr. Kirkbride,

Thank you very much for submitting your manuscript "A nationwide study of the incidence of substance use disorders in refugee and migrant groups in Sweden: a cohort study of 1.2 million people" (PMEDICINE-D-19-02064) for consideration for our upcoming special issue on substance mis/use at PLOS Medicine.

Your paper was discussed among the editorial team, evaluated by the guest editors for the special issue, and sent to independent reviewers, including a statistical reviewer. The reviews are appended at the bottom of this email and any accompanying reviewer attachments can be seen via the link below:

In light of these reviews, we will not be able to accept the manuscript for publication in the journal in its current form, but we would like to invite you to submit a revised version that fully addresses the reviewers' and editors' comments. The questions raised by referee 2 about valid outcome ascertainment and possible selection seem important to us.

You will appreciate that we cannot make a decision about publication until we have seen the revised manuscript and your response, and we expect to seek re-review by one or more of the reviewers.

In revising the manuscript for further consideration, your revisions should address the specific points made by each reviewer and the editors. Please also check the guidelines for revised papers at <https://eur01.safelinks.protection.outlook.com/?url=http%3A%2F%2Fjournals.plos.org%2Fplosmedicine%2Fs%2Frevising-your-manuscript&amp;data=02%7C01%7C%7Ced0a6866261e4f4a68d908d710f13271%7C1faf88fea9984c5b93c9210a11d9a5c2%7C0%7C0%7C636996500428902822&amp;sdata=xxGc13Iuto%2F4utYS7iJgtIBzVtWlDhZPTkqv5xlDytA%3D&amp;reserved=0> for any that apply to your paper. In your rebuttal letter you should indicate your response to the reviewers' and editors' comments, the changes you have made in the manuscript, and include either an excerpt of the revised text or the location (eg: page and line number) where each change can be found. Please submit a clean version of the paper as the main article file; a version with changes marked should be uploaded as a marked up manuscript.

**We have followed these instructions**

In addition, we request that you upload any figures associated with your paper as individual TIF or EPS files with 300dpi resolution at resubmission; please read our figure guidelines for more information on our requirements: <https://eur01.safelinks.protection.outlook.com/?url=http%3A%2F%2Fjournals.plos.org%2Fplosmedicine%2Fs%2Ffigures&amp;data=02%7C01%7C%7Ced0a6866261e4f4a68d908d710f13271%7C1faf88fea9984c5b93c9210a11d9a5c2%7C0%7C0%7C636996500428902822&amp;sdata=Yyi8mKD3hGPP8tQRstEy6lzYRqA5dBzt3cEyUPlPV%2Bs%3D&amp;reserved=0>. While revising your submission, please upload your figure files to the PACE digital diagnostic tool, <https://eur01.safelinks.protection.outlook.com/?url=https%3A%2F%2Fpacev2.apexcovantage.com%2F&amp;data=02%7C01%7C%7Ced0a6866261e4f4a68d908d710f13271%7C1faf88fea9984c5b93c9210a11d9a5c2%7C0%7C0%7C636996500428902822&amp;sdata=ooKfMLNhqwqHd4tQJb4pVuQfzoRECiZQYZjL7g%2BJ9UI%3D&amp;reserved=0>. PACE helps ensure that figures meet PLOS requirements. To use PACE, you must first register as a user. Then, login and navigate to the UPLOAD tab, where you will find detailed instructions on how to use the tool. If you encounter any issues or have any questions when using PACE, please email us at [PLOSMedicine@plos.org](mailto:PLOSMedicine@plos.org).

**We have followed these instructions**

We hope to receive your revised manuscript within two weeks. Please email us ([plosmedicine@plos.org](mailto:plosmedicine@plos.org)) if you have any questions or concerns.

***Please note while forming your response, if your article is accepted, you may have the opportunity to make the peer review history publicly available. The record will include editor decision letters (with reviews) and your responses to reviewer comments. If eligible, we will contact you to opt in or out.***

We ask every co-author listed on the manuscript to fill in a contributing author statement, making sure to declare all competing interests. If any of the co-authors have not filled in the statement, we will remind them to do so when the paper is revised. If all statements are not completed in a timely fashion this could hold up the re-review process. If new competing interests are declared later in the revision process, this may also hold up the submission. Should there be a problem getting one of your co-authors to fill in a statement we will be in contact. YOU MUST NOT ADD OR REMOVE AUTHORS UNLESS YOU HAVE ALERTED THE EDITOR HANDLING THE MANUSCRIPT TO THE CHANGE AND THEY SPECIFICALLY HAVE AGREED TO IT. You can see our competing interests policy here: <https://eur01.safelinks.protection.outlook.com/?url=http%3A%2F%2Fjournals.plos.org%2Fplosmedicine%2Fs%2Fcompeting-interests&amp;data=02%7C01%7C%7Ced0a6866261e4f4a68d908d710f13271%7C1faf88fea9984c5b93c9210a11d9a5c2%7C0%7C0%7C636996500428902822&amp;sdata=TgSdEgx6lduuR1dL7XokMt4zg84Tw5mhv6ieq8izYY4%3D&amp;reserved=0>.

Please use the following link to submit the revised manuscript:

<https://eur01.safelinks.protection.outlook.com/?url=https%3A%2F%2Fwww.editorialmanager.com%2Fpmedicine%2F&amp;data=02%7C01%7C%7Ced0a6866261e4f4a68d908d710f13271%7C1faf88fea9984c5b93c9210a11d9a5c2%7C0%7C0%7C636996500428912819&amp;sdata=sLHkouEweSXhrIKhqGS4dX87zJ4jsEhtAOZ6TOz5Yp8%3D&amp;reserved=0>

Your article can be found in the "Submissions Needing Revision" folder.

To enhance the reproducibility of your results, we recommend that you deposit your laboratory protocols in protocols.io, where a protocol can be assigned its own identifier (DOI) such that it can be cited independently in the future. For instructions see <https://eur01.safelinks.protection.outlook.com/?url=http%3A%2F%2Fjournals.plos.org%2Fplosmedicine%2Fs%2Fsubmission-guidelines%23loc-methods&amp;data=02%7C01%7C%7Ced0a6866261e4f4a68d908d710f13271%7C1faf88fea9984c5b93c9210a11d9a5c2%7C0%7C0%7C636996500428912819&amp;sdata=aEY82%2Bgzfkt0tRk7LlL%2FjqOxsS2JglOWHHeiQeq9TrU%3D&amp;reserved=0>.

**We have done so. Please see protocols.io (**[**dx.doi.org/10.17504/protocols.io.53fg8jn**](https://dx.doi.org/10.17504/protocols.io.53fg8jn)**).**

Please ensure that the paper adheres to the PLOS Data Availability Policy (see <https://eur01.safelinks.protection.outlook.com/?url=http%3A%2F%2Fjournals.plos.org%2Fplosmedicine%2Fs%2Fdata-availability&amp;data=02%7C01%7C%7Ced0a6866261e4f4a68d908d710f13271%7C1faf88fea9984c5b93c9210a11d9a5c2%7C0%7C0%7C636996500428912819&amp;sdata=Np2xPhjhuL5q2aXqxTziGyz1EDDtcgMu1re01153LCE%3D&amp;reserved=0>), which requires that all data underlying the study's findings be provided in a repository or as Supporting Information. For data residing with a third party, authors are required to provide instructions with contact information for obtaining the data. PLOS journals do not allow statements supported by "data not shown" or "unpublished results." For such statements, authors must provide supporting data or cite public sources that include it.

**We have provided a data sharing statement in the manuscript (P23, lines 554-562). Under ethical approval and Swedish law we do not have a legal basis to make the data publicly available as the data resides with a third party. Nonetheless, we have provided full contact details, in English and Swedish, about how to obtain the data, and have made our analysis scripts freely available at PsyArXiv and provided the link to this address.**

Please let me know if you have any questions. Otherwise, we will look forward to receiving your revised manuscript shortly.

Sincerely,

Richard Turner PhD, for Philippa Berman, MBBS Senior Editor, PLOS Medicine

[rturner@plos.org](mailto:rturner@plos.org)

-----------------------------------------------------------

Requests from the editors:

For researchers who wish to inquire about data access but are unfamiliar with Swedish, please ensure that you provide well-signposted non-author contact details.

**We have revised the Data Sharing section to give details of the PI of the Psychiatry Sweden project, who can advise on data sharing in English or Swedish. See P23 (lines 561-562)**

You may wish to restructure the title, e.g. "Substance use disorders in refugee and migrant groups in Sweden: a nationwide study of 1.2 million people".

**We are happy with the suggested title revision and have re-written it accordingly (P1, lines 6-8).**

Please add some additional details to your abstract, e.g. quoting the number of refugees; summary demographic details for study participants; and the major countries or regions contributing refugees and/or migrants.

**We have added these summary details in the abstract, P2, lines 49-52**

Please add p values alongside CI where available.

**We have added these details throughout the manuscript.**

Please avoid the phrase "refugee generating".

**We have changed the wording on P5 (lines 112-114) to now read:**

**“For example, one study in Sweden found that hospitalizations for alcohol and drug use disorders were lower in immigrants from regions of the world where Sweden has traditionally received substantial numbers of refugees, compared with the Swedish-born population [18]. This study, however, did not have a direct measure of refugee status.”**

**And on P20, lines 493-494:**

**“As we wanted to ensure valid comparisons between refugee and non-refugee migrants, we restricted our sample to participants from regions of the world where Sweden has traditionally received substantial refugee flows.”**

The final sentence of the "methods and findings" subsection of your abstract should summarize the study's main limitations.

**We have corrected the abstract to include a “methods and findings” section and added the main limitation of the study to that section to read (P3, lines 62-65):**

**“Our findings highlight differences in the diagnosed incidence of substance use disorders detected in secondary and emergency care in Sweden, as recorded in the Swedish National Patient Register, but may have led to non-differential or differential under-ascertainment (by migrant status) of those only seen via primary care.”**

After your abstract, we will need to ask you to add a new and accessible "author summary" section in non-identical prose. You may find it helpful to refer to one or two recent research papers in PLOS Medicine to get a sense of the preferred style.

**We have added this section. See P4**

Please restructure the text around lines 95-105 to create a shorter section stating the study's aims. Where you discuss expectations, please make these points earlier in the introduction, supported by references as appropriate.

**We have now restructured the text in the introduction to be clearer, introducing the expectations earlier in the section (i.e. P6-7, lines 122-129, 138, 142-174).**

Early in your methods section, please state whether or not the study had a protocol or prespecified analysis plan, and if so attach the document as a supplementary file. Please identify any analyses that were not prespecified.

**We have added the protocol original developed for the study for Ms. Samantha Harris’ dissertation on the M.Sc. in Clinical Mental Health Sciences at UCL (see S1 Appendix); the submitted manuscript is based on that protocol. We have also added the protocol to protocols.io (****[dx.doi.org/10.17504/protocols.io.53fg8jn](https://dx.doi.org/10.17504/protocols.io.53fg8jn)). Finally, we have updated the manuscript to state this and to state any analyses (as suggested by the reviewers, below) which were not part of the original protocol (P8, lines 176-178).**

In the discussion section of your main text, please craft a discrete paragraph or paragraphs summarizing the study's limitations.

**We have revised the Strengths and Limitations section (P17-19) to have separate paragraphs on our limitations, including additional discussion raised in response to the reviewers.**

Please ensure that all references comply with journal style. We noted that journal names appeared to be missing for some references (e.g. references 6 and 10). Generally, publisher names can be removed (e.g. for reference 8). Referee 29 seems to lack full access details.

**We apologise and have attended to these inconsistencies, which have been corrected (see References, P24-27).**

We would suggest making the participant flowchart, supplementary figure 1, part of the main paper.

**We have done this and renumbered all figures accordingly.**

Please make your STROBE checklist a separate supplementary file. Individual items should be referred to by section (e.g. "methods") and paragraph number rather than by page or line numbers, as the latter generally change in the event of publication.

**We have followed these instructions and moved the STROBE checklist to “S2 Appendix” and renumber other supplementary tables accordingly throughout the manuscript.**

Comments from the reviewers:

*** Reviewer #1:

The paper "A nationwide study of the incidence of substance use disorders in refugee and migrant groups in Sweden: a cohort study of 1.2 million people" examines whether risk of substance use disorders varies by region of origin and diagnosis of post-traumatic stress disorder. The authors find lower rates of substance use disorders in migrants and refugees and that a PTSD diagnosis was associated with substance use disorder.

*	The paper examines an important research question. However, I find the paper too descriptive and the results does not therefore contribute significantly to the progression of knowledge within this field of research.

**Thank you for this important point. To strengthen the analytic approach, we have added analyses by age-at-migration and time lived in Sweden (please see Table 4, and supplemental tables S4-S6). The main changes to our paper are described in brief below, but we consider these additional analyses have brought welcome new insights, most notably strong dose-response evidence of convergence to the rate of substance use disorders in the Swedish-born population over time (whether measured by age-at-migration or time in Sweden).**

**We used categorical definitions of both these exposures. Given that we have now included four exposure variables (migrant status, region of origin, age-at-migration and time in Sweden), all of which had the same baseline or reference category (i.e. the Swedish-born population) we had to consider the substantive issue of multicollinearity between these variables. This meant we could not mutually adjust models for all four exposures simultaneously in the full dataset. To overcome this issue, we modelled each exposure separately (in unadjusted and adjusted models), reporting Akaike’s Information Criterion (AICs) for each model (S5 Table), where a lower score indicated better model fit.**

**For all substance use disorders as an outcome, we also ran our adjusted model on a sub-sample restricted to the migrant sample, excluding the Swedish-born population, allowing us to overcome the multicollinearity issue and mutually adjust for these four exposures simultaneously (S6 Table). These indicated independent effects of region-of-origin, age-at-migration and time in Sweden.**

**The results of these revised and extended analyses confirmed our updated hypotheses (P7, lines 159-160) that rates of substance use disorders in migrants would become more similar to the rate in the Swedish born population over time. In particular, we observed the following results:**

- **First, earlier age-at-migration and longer time in Sweden increased risk, with rates converging towards the Swedish-born rate over time in a dose-dependent manner. Thus, for all outcomes, no differences in substance use disorders were observed in migrants who had lived in Sweden for 10 or more years than the Swedish-born population.**
- **Second, for any SUD age-at-migration was the best predictor to the data (S6 Table), and joint best with time in Sweden for alcohol use disorders. It also performed second best for cannabis use and polydrug use disorders, behind region of origin.**
- **Third, in our revised analysis, there was evidence that adjusted hazard ratios for cannabis use disorders in the sub-Saharan African group were higher than in the Swedish-born population (HR: 1.40; 95%CI: 1.11-1.75), not equivocal as previously reported. Cannabis use disorders also showed less reduced risk in non-refugee migrants (HR: 0.81; 95%CI: 0.70-0.93) and refugees (HR: 0.96; 95%CI: 0.72-1.29) and we have revised the manuscript accordingly.**
- **Fourth, our revised analysis led to a change in results of effect modification between migrant status and PTSD on all substance use disorder outcomes, which all met statistical significance, as updated in a revised table (Table 5) and reported in the results and discussion. In brief, while the presence of PTSD generally led to a marked increase in risk of all substance use disorders for refugees, non-refugee migrants and the Swedish-born groups, this effect was more pronounced amongst Swedish-born participants, particularly for alcohol and cannabis use disorders. In line with our interpretation of other findings in our revised discussion, we suggest this may be attributable to cultural differences in behaviours and attitudes towards substance use (P20, lines 485-486).**

**We have updated the results with a new section in the text on age-at-migration and time in Sweden (P14-15, lines 345-363; Table 4, S4-S6 tables). We have also added methods for these variables (P9, lines 222, 225-228) and how they were handled in the revised statistical analyses (P11, lines 259-262, 267-270). Finally, we have added discussion of these results on P16 (lines 398-405) and P21 (lines 521-530).**

**We believe these additional results now provide considerable further insight offered by our paper in this large, nationwide cohort of incidence substance use disorders.**

*	The authors argue that one of the important contributions of the paper is that they study risks of substance use disorders by region of origin. However the region or origin groups are too crude to give any additional insight in how country specific and culture specific factors could have influenced the findings. Especially the group of migrants from "Asia, Middle East and North Africa" is extremely broad and includes many different cultures, religions and nationalities. Therefore it is difficult to make any meaningful interpretations from the results.

**We have now acknowledged that the region-of-origin categories are broad in our limitations section (P19, lines 471-474). For the benefit of the reviewer, please note that Asia was considered separately to the “Middle East and North Africa” – we have changed the punctuation on P9 (line 223) to clarify this. Our broad region-of-origin categories were a function of the sample size, which although large in the context of cohort studies of psychiatric disorders, did not permit finer-grained analysis by narrower regions of origin. Further, the Swedish Migrancy Agency does not disaggregate register data by every single country of origin.**

**To aid interpretation of the data by region of origin, we have added a supplemental table (S1 Table) which provides additional information about the narrower regions of origin and countries of origin in our cohort. This data shows that the majority of our migrant sample came from specific parts of Africa (East Africa – 10.3%, specifically Somalia, Eritrea, Ethiopia and Djibouti), Asia (Central Asia – 10.8%, including Afghanistan), Eastern Europe (10.1%), countries within the former Yugoslavia (10.2%) and the Middle East (specifically, Iraq – 24.5%). We have added narrative discussion of this to the methods, results and discussion sections of the manuscript.**

*	It is highly unclear why the authors decided to follow a cohort born between 1984 and 1997. These are fairly young immigrants. This should be explained much better in the paper.

**The reviewer is correct that this sample is relatively young (up to 32 years old). We restricted our cohort to this age period to ensure that we could follow a birth cohort from their 14^th^ birthday until a diagnosis of substance use disorder diagnosed under ICD-10 only (first introduced in Sweden in 1997), and not ICD-9, ICD-8 etc. We have added a line to this effect in the methods (P8, lines 184-188).**

*	It would have been interesting to separately to study the group of migrants arriving without parents separately.

**This is an interesting issue but was beyond the scope of our original aims, and the numbers would likely have been low, even in a large cohort.**

*	The findings does not really contribute to any new insights. We already know that many groups of migrants consume less alcohol and drugs. The paper would have been much more interesting if the analyses included information on duration of residence in Sweden and age at migration. Such variables could give important additional knowledge on migrants and refugees drinking patterns.

**Please see our response to your first comment.**

*	Some of the hypotheses are poorly explained. Why do the authors expect that refugees but not non-refugees have a higher risk of substance disorder? Why do the authors expect that those with PTSD diagnosis will have higher rates of substance disorders? What is the main explanation here?

**We have clarified our aims and hypotheses in the introduction following editorial suggestion (P6-7), and hope this has now clarified our views. We expected refugees would have higher rates of substance use disorders because they are more likely to have been exposed to traumatic life events and meet diagnosis for PTSD; PTSD is strongly associated with substance abuse disorders. While we found higher levels of PTSD amongst refugees than other groups, in partial support of this hypothesis, the effect of PTSD on substance use disorders was most pronounced in the Swedish-born group (Table 5) in contrast to our hypothesis of no effect modification, and we discuss this on P20, lines 485-486 in light of the reviewer’s suggestion below that cultural norms and behaviors may be more relevant.**

*	It is unclear why the authors primarily suggest that the findings could be explained by the healthy migrant effect. I believe that substance use disorders are highly related to culture and religion and substance use is much lower in many countries outside Sweden. Therefore, the most likely explanation is not that migrants constitute a selection of "healthier" individuals with regard to substance use. It is rather that consumption patterns in the country of origin are lower for different reasons.

**We have re-phrased this section of the discussion to give more prominence to acknowledge cultural/religious factors which might affect patterns of composition, with less emphasis on the healthy migrant effect as the “main” explanation. We think this section now has much better balance and thank the reviewer. See P21, lines 508-521:**

**“Lower rates of substance use disorders – which we observed to a similar extent in both refugee and non-refugee migrant groups from the same regions of origin – may be attributable to various factors, including the ‘healthy immigrant effect’ or sociocultural and religious differences in attitudes and behaviours towards substance use. The healthy migrant effect suggests that people who chose to migrate are more motivated, adaptive, often younger and therefore healthier than the native-born population. However, such an effect may be less likely to explain lower rates of substance use disorders in refugee~~s~~, although it has been posited [21] that they may benefit from additional institutional and contextual levers in their country of settlement which provide further support against harmful behaviours [21,22].**

**Alternatively, lower rates of substance use disorders we observed for refugee and non-refugee migrants compared with the Swedish-born population may be attributable to cultural norms or religious views on substance use behaviours.”**

*	Undocumented migrants and refugees probably have much higher levels of substance use. This should be acknowledged or at least discussed in the paper.

**Although we intuitively agree with the reviewer, we are unaware of any empirical evidence to support this statement so have not included it in this revision. Undocumented groups were not part of our cohort study, and we have clarified our statement about this in the methods on P8, line 193:**

**“Those not officially granted residence in Sweden (i.e. asylum seekers and undocumented migrants)…were excluded.”**

**We have now stated that our findings may not generalize to these groups in the limitations section (P19, lines 473-474):**

**“Our findings may not generalize to asylum seekers or undocumented migrants who were not included in the present study.”**

*	The restriction of the sample to participants from major refugee-generating regions should be explained earlier.

**We have added a line to this effect in the introduction (P7, lines 153-154):**

**“We therefore investigated whether the incidence of substance use disorders in refugees and non-refugees migrants from regions with substantial refugee flows to Sweden differed to the native-born population…”**

*** Reviewer #2:

There are two main problems with the methods that mean that this study's findings are difficult to interpret.

The first is that there is no evidence cited on the validity of the particular outcomes (substance use disorders and PTSD) in the study. Are substance use disorder diagnoses valid in these registers? Does this validity extend to subcategories of substance use disorders, such as a cannabis use disorder. My impression is that these Nordic registers have good validity for severe mental illness, but this does not extend to other diagnoses, including PTSD and substance use subcategories.

**This is a very important question and we thank the reviewer for highlighting it. In general the Swedish National Patient register is known to be valid for psychiatric diagnoses (Ludvigsson et al 2011, but this has not yet been specifically investigated for substance use disorders. Further, our reliance on case ascertainment from secondary sources (raised as a limitation below) is a strength in this context, given that diagnoses will have been made in inpatient care for substance use disorders or specialized alcohol and substance use outpatient clinics, by specialized physicians working in this area. We believe this lends some confidence to the likely validity of these diagnoses, notwithstanding the pressing need for purposeful validation studies. Some co-authors (ACH, CD) have a paper under review which shows good validity of PTSD diagnoses in the register. We have referenced this paper (Hollander et al, under review) in the manuscript and will update details as soon as it is in press. We have discussed these issues as an important limitation of the data in a revised limitations section (see P18-19, lines 456-461).**

**References:**

**Hollander A-C, Askegård K, Escalante C, Holmes EA, Wicks S, Dalman C. A validation study of cases of PTSD diagnoses identified in a Swedish regional database - Is the validity sufficient for epidemiological research? Under Review.**

**Ludvigsson JF, Andersson E, Ekbom A, Feychting M, Kim JL, Reuterwall C, et al. External review and validation of the Swedish national inpatient register. BMC Public Health. 2011;11: 450. doi:10.1186/1471-2458-11-450**

A second problem is that the authors have used predominately inpatient information to calculate prevalence rates - with outpatient diagnoses added from 2005. The problem here is that most substance use disorders are diagnosed in primary care (i.e. not outpatients) and therefore this study will have missed the majority of substance use disorders. This is borne out in the prevalence rates which are very low compared to other epidemiological surveys. In the current study, alcohol is estimated at an incidence of 208 per 100,000 person years, which seems around 10x lower than expected. The same applies for substance use disorders (which is reported at 290 per 100,000 person years). Some comparison of these incidence rates with other surveys, including the GBD would be helpful.

**We thank the reviewer for raising this important concern. All studies of psychiatric disorders based on Swedish register data suffer from similar issues of case ascertainment, and we acknowledge the careful need to interpret our findings in this light. The salient issue here appears to be the lack of coverage of primary care contacts, not the distinction between in- or out-patient case ascertainment. Primary care records are not currently available in the Swedish registers. We acknowledge this will have led to some non-differential case ascertainment resulting in an underestimate of the true incidence of substance use disorders in Sweden. Nonetheless, our results provide useful information about the *treated* incidence of substance use disorders in Sweden that require secondary care, either via in-patient admission or specialized outpatient care, and highlight differences between the Swedish-born and migrant populations in this regard. It is less clear whether differential case ascertainment bias by migrant status will have affected our results with respect to reliance on secondary care contacts. In general, migrant groups may have worse healthcare literacy or more reluctance to engage with any state-provided healthcare. This would lower their universal contact with services, although may increase their probability of being detected in secondary care, if for example, their substance use issues became more severe as a result of lack of engagement with primary care. Rightly, we have revised our limitations section to give this issue much greater prominence (P17-18, lines 428-441).**

**Our study did include 100% nationwide coverage of inpatient data over 19 years of the follow-up period, 100% coverage of out-patient care for 11 of those 19 years and some level of coverage of outpatient care for a further 5 years (between 2001-2005) – although due to methodological reasons around how the Swedish registers were implemented it is not possible to establish the exact proportion of outpatient care covered in this period (see Ludvigsson et al 2011, above). Before 2001, outpatient care was not included in the Swedish registers, but specialized psychiatric outpatient care was also less common at this point in time than it is today, with most psychiatric care based on inpatient admissions. We therefore believe that the lack of outpatient coverage between 1998-2000 is a relatively minor issue compared with the issues of validity and lack of primary care coverage in the registers, as discussed above.**

**Finally, we are grateful to the reviewer for questioning the reported rates of substance use disorder in our study, but do not believe that the incidence rates (number of new cases per 100,000 people per year) we report are in fact substantially low. We think the reviewer may have conflated these figures with prevalence which they discuss interchangeably in this review point (proportion of new and existing cases in a population at a given time point or period), on which most earlier research has relied. We found the incidence rates of any substance use disorder (identified via secondary care) in the Swedish-born population to be 290 new cases per 100,000 people per year. In the same population, the comparable figure for non-affective psychotic disorders is 38.5 (7.5 times smaller). This illustrates the magnitude and severity of this public health issue, which we have commented on in the discussion (P18, lines 434-437). We would expect prevalence to be higher, because it includes new and existing cases, but prevalence was not the measure of disease frequency of interest in this cohort study. We hope that clarifies this concern.**

These two problems may not differentially affect refugees and non-migrant refugees, but as the authors suggest in the limitations, such populations are less likely to access healthcare services. This will likely be more an issue for inpatient services, and therefore the findings of the relative differences between the groups may be biased. Furthermore, there may be more stigma in receiving a substance use disorder diagnosis, particularly alcohol (as it is against the Muslim religion and most refugees to Sweden will be from Islamic countries), which would further explain the reported differences.

**We have commented on these issues at length in our discussion, including the role of stigma, and expanded on this issue (P17-18, lines 441-427; p21, lines 528-530).**

*** Reviewer #3 (statistical reviewer):

This is a well-conducted and comprehensive study on the incidence of substance use disorders in refugee and migrant groups in Sweden. The study design, datasets, statistical methods and analyses, and presentation (tables and figures) and interpretation of results are mostly adequate and of a high standard. Missing data and proportional hazards assumption are two tricky issues but were dealt with very well by the authors in both analyses and discussion, well done!

**Thank you for this very positive and reassuring assessment of our paper.**

Only a few minor points needing attention:

1) Page 10, Line 217-219, apart from the crude incidence of SUD, can authors also provide age- and sex-adjusted incidence rates?

**After careful consideration, we have chosen to omit age-sex standardized rates of SUDs in the manuscript. The crude rates provide an indication of the unadjusted treated incidence of various SUDs in this Swedish cohort, and the adjusted hazard ratios we present already include adjustment for age and sex, as well as several other covariates included in the models. Following requests from other reviewers to expand the paper to provide more analytical insights, we felt that age-sex standardization would have only added further descriptive (rather than analytical) results to the paper.**

2) Can authors provide death data for the cohorts by groups? Assume very low as it's a young cohort but just to want to make sure there is no competing risk issue in applying cox models when outcomes are not all-cause mortality.

**The absolute mortality rate in this sample was low (less than 19 deaths per 100,000 people per year) and we do not believe would have affected our results as a competing risk. We have added a supplementary table (S8 Table) to show this and added a brief comment to the limitations (P17, lines 424-427):**

**“Rates of all-cause mortality in this young sample were low (S5 Table) and were therefore unlikely to have acted as a competing risk on our results.”**

3) Page 6, Line 110, "Using linked, longitudinal Swedish register data from Psychiatry Sweden". By the way, any brief explanation or reference for "Psychiatry Sweden"?

**Thank you and apologies. We have clarified what “Psychiatry Sweden” is in the text on P8, lines 181-182, as:**

**“Using linked, longitudinal Swedish register data from Psychiatry Sweden, a database of linked registers to explore the causes and outcomes of mental health disorders, we established an initial cohort…”**

***

Any attachments provided with reviews can be seen via the following link:

__________________________________________________

In compliance with data protection regulations, you may request that we remove your personal registration details at any time. (Use the following URL: <https://eur01.safelinks.protection.outlook.com/?url=https%3A%2F%2Fwww.editorialmanager.com%2Fpmedicine%2Flogin.asp%3Fa%3Dr&amp;data=02%7C01%7C%7Ced0a6866261e4f4a68d908d710f13271%7C1faf88fea9984c5b93c9210a11d9a5c2%7C0%7C0%7C636996500428912819&amp;sdata=vRpK8kXPuOA8xnjOWKTzS4BQmFJ1GlSdGddUGR7DDw8%3D&amp;reserved=0>). Please contact the publication office if you have any questions.
