# Supplementary material for: Substance use disorders in refugee and migrant groups in Sweden: A nationwide cohort study of 1.2 million people
Source: PLoS Med. 2019 Nov 5;16(11):e1002944. doi: 10.1371/journal.pmed.1002944 (PMC6830745; doi:10.1371/journal.pmed.1002944)
Supplement: Attachment — Submitted filename: Response to reviewers PLoS Medicine R1.docx [file pmed.1002944.s013.docx]

**Author note: all changes have been made as indicated below. Line and page numbers refer to the fully tracked version of the manuscript. We have separated our comments into those from the ”Editorial and reviewer comments” email and those from the ”Production” email received from PloS Medicine. We are grateful for your time.**

**Editorial and reviewer comments**

**From:** PLOS Medicine <[em@editorialmanager.com](mailto:em@editorialmanager.com)>
**Sent:** Wednesday, 4 September 2019 15:47
**To:** Kirkbride, James
**Subject:** PMEDICINE-D-19-02064R1 Decision - [EMID:57bdbefee950a2ad]

CC: [rturner@plos.org](mailto:rturner@plos.org)

Dear Dr. Kirkbride,

Thank you very much for re-submitting your manuscript "Substance use disorders in refugee and migrant groups in Sweden: a nationwide cohort study of 1.2 million people" (PMEDICINE-D-19-02064R1) for consideration at PLOS Medicine for our upcoming special issue on substance mis/use.

I have discussed the paper with editorial colleagues and our academic editor, and it was also seen again by three external reviewers. I am pleased to tell you that, provided the remaining editorial and production issues are dealt with, we expect to be able to accept the paper for publication in the journal.

The remaining issues that need to be addressed are listed at the end of this email. Any accompanying reviewer attachments can be seen via the link below. Please take these into account before resubmitting your manuscript:


Our publications team ([plosmedicine@plos.org](mailto:plosmedicine@plos.org)) will be in touch shortly about the production requirements for your paper, and the link and deadline for resubmission. DO NOT RESUBMIT BEFORE YOU'VE RECEIVED THE PRODUCTION REQUIREMENTS.

***Please note while forming your response, if your article is accepted, you may have the opportunity to make the peer review history publicly available. The record will include editor decision letters (with reviews) and your responses to reviewer comments. If eligible, we will contact you to opt in or out.***

In revising the manuscript for further consideration here, please ensure you address the specific points made by each reviewer and the editors. In your rebuttal letter you should indicate your response to the reviewers' and editors' comments and the changes you have made in the manuscript. Please submit a clean version of the paper as the main article file. A version with changes marked must also be uploaded as a marked up manuscript file.

Please also check the guidelines for revised papers at [https://eur01.safelinks.protection.outlook.com/?url=http%3A%2F%2Fjournals.plos.org%2Fplosmedicine%2Fs%2Frevising-your-manuscript&amp;data=02%7C01%7C%7C9f5621fbe08a42fadfdc08d73146cdcf%7C1faf88fea9984c5b93c9210a11d9a5c2%7C0%7C0%7C637032052471424603&amp;sdata=m0GZ5QahhqvCZdAW6%2Bw9uI8%2FJ68q9F8yZLcZ%2BzGPhLw%3D&amp;reserved=0](https://eur01.safelinks.protection.outlook.com/?url=http%3A%2F%2Fjournals.plos.org%2Fplosmedicine%2Fs%2Frevising-your-manuscript&data=02%7C01%7C%7C4859ae2d453a40d130e608d731d6e828%7C1faf88fea9984c5b93c9210a11d9a5c2%7C0%7C0%7C637032671399705168&sdata=vjlJDIwFs1jOPjvYGnSuHzyfPVSn4%2BidHVOGLpCAWgI%3D&reserved=0) for any that apply to your paper. If you haven't already, we ask that you provide a short, non-technical Author Summary of your research to make findings accessible to a wide audience that includes both scientists and non-scientists. The Author Summary should immediately follow the Abstract in your revised manuscript. This text is subject to editorial change and should be distinct from the scientific abstract.

We hope to receive your revised manuscript within one week. Please email us ([plosmedicine@plos.org](mailto:plosmedicine@plos.org)) if you have any questions or concerns.

We ask every co-author listed on the manuscript to fill in a contributing author statement. If any of the co-authors have not filled in the statement, we will remind them to do so when the paper is revised. If all statements are not completed in a timely fashion this could hold up the re-review process. Should there be a problem getting one of your co-authors to fill in a statement we will be in contact. YOU MUST NOT ADD OR REMOVE AUTHORS UNLESS YOU HAVE ALERTED THE EDITOR HANDLING THE MANUSCRIPT TO THE CHANGE AND THEY SPECIFICALLY HAVE AGREED TO IT.

Please ensure that the paper adheres to the PLOS Data Availability Policy (see [https://eur01.safelinks.protection.outlook.com/?url=http%3A%2F%2Fjournals.plos.org%2Fplosmedicine%2Fs%2Fdata-availability&amp;data=02%7C01%7C%7C9f5621fbe08a42fadfdc08d73146cdcf%7C1faf88fea9984c5b93c9210a11d9a5c2%7C0%7C0%7C637032052471424603&amp;sdata=Ox0rxBlf0qcZbHflxpeSZShLYz1uS%2F7YAVNmE%2BpxcyI%3D&amp;reserved=0](https://eur01.safelinks.protection.outlook.com/?url=http%3A%2F%2Fjournals.plos.org%2Fplosmedicine%2Fs%2Fdata-availability&data=02%7C01%7C%7C4859ae2d453a40d130e608d731d6e828%7C1faf88fea9984c5b93c9210a11d9a5c2%7C0%7C0%7C637032671399715164&sdata=r0NSqfb%2Bqzlz%2FmOHxOePwWa6%2BIE%2F0h0mSVlmySI8zKk%3D&reserved=0)), which requires that all data underlying the study's findings be provided in a repository or as Supporting Information. For data residing with a third party, authors are required to provide instructions with contact information for obtaining the data. PLOS journals do not allow statements supported by "data not shown" or "unpublished results." For such statements, authors must provide supporting data or cite public sources that include it.

Please let me know if you have any questions. Otherwise, we will look forward to receiving the revised manuscript soon.  

Kind regards,

Richard Turner PhD, for Philippa Berman, MBBS
Senior Editor, PLOS Medicine

[rturner@plos.org](mailto:rturner@plos.org)

------------------------------------------------------------

Requests from Editors:

Please remove Prof Dalman's contact from your data statement, as she is an author on the paper, and substitute an alternative contact who is not an author.

**We have done so and replaced this with an appropriate contact details for the Swedish Research Council (in English). This addition now reads:**

**”Researchers can also contact the Swedish Research Council about how to access register-based data by visiting https://www.registerforskning.se/en/ or contacting them by email (registerforskning@vr.se) or telephone +46 8 546 440 00.”**

**In accordance with production requirements (see below) this section has been removed from the manuscript, but updated in the “Data availability” field in the submission form**

Please revisit the phrase "import healthier behaviours" (abstract and author summary). We suggest substituting a phrase reflecting the behaviours prevalent in migrants' countries of origin.

**We have changed this to ”...lower rates of substance use disorders in migrants and refugees may reflect prevalent behaviors with respect to substance use in migrants’ countries of origin...” P3 and ”Prevalent behaviors with respect to substance use in refugees and migrants...may limit...” P4**

Around line 48, where you mention the regions of origin of migrant and refugee groups, we suggest revising the text to "Refugees' regions of origin were represented in proportions ranging from 6.0% (Eastern Europe & Russia) to 41.4% (Middle East and North Africa); Proportions of non-refugee migrants' regions of origin ranged from 11.8% (sub-Saharan Africa) to 33.7% (Middle East and North Africa).", or similar.

**We have used this wording and revised the abstract accordingly. Lines 48-50, P2.**

At line 55, you may wish to adapt "... and for migrants from most regions-of-origin" to "and the findings did not differ substantially by migrants' region of origin.".

**We have used this wording and revised the abstract accordingly. Lines 58-59, P3.**

At lines 57 and 87, considering the observational design of your study we ask you to substitute "was more strongly associated with", or similar, for "had more pronounced effects". Please also adapt any other instances of language ascribing causality in the ms.

**We have used this wording and revised the abstract accordingly. Line 60, P3. We have replaced similar instances in the manuscript.**

The sentence at line 59 ("Our findings highlight ... ) would seem more suitable for the "Conclusions" subsection of your abstract.

**We included this statement in ”Methods and findings” to adhere to the instructions to authors that this section should ”Describe the main limitations of the study.” (**[**https://journals.plos.org/plosmedicine/s/submission-guidelines**](https://journals.plos.org/plosmedicine/s/submission-guidelines)**). In light of the next editorial comment, we have retained it here, but revised the wording as required below. P3.**

Please adapt the final sentence of the "Methods and Findings" subsection of your abstract so that it begins "The main limitations of our study were possible differential or non-differential under-ascertainment (by migrant status) of those only seen in primary care.". Please add one further limitation to this sentence, which might focus on the unknown status of undocumented refugees and migrants, for example.

**We have attended to these requirements. We have added a sentence on undocumented refugees and migrants (who are not part of this cohort). P3, lines 66-67.**

At line 63, please start the sentence with "Our findings suggest that ..." or similar.

**We have done so.** **Line 68, P3.**

At line 150, please correct "be were lower".

**We have done so.** **Line 155, P7.**

Around lines 150-155, please remove the bullet points and convert this into narrative text, separating the relevant points with semicolons as needed.

**We have done so. Changes not tracked for ease of reading this revised section but wording remains identical except for the removal of bullets. Lines 155-160, P7.**

Please check journal name abbreviations in your reference list, and make any corrections needed (e.g. "CMAJ" rather than "Cmaj")

**We have done so.** **P26, P28.**

Are you able to add a URL for reference 41? Please include an accessed date if so.

**We have done so. Now reference 43.** **P29.**

Please remove reference 44, unless this is available as a preprint; or if the paper is "in press" (if so, please send us the acceptance letter).

**Unfortunately this paper remains under review and so we have removed it from this manuscript along with the corresponding sentence on lines 454-455, P21.**

Comments from Reviewers:

*** Reviewer #1:

I am satisfied with the revisions made by the authors. I especially believe that considering age at migration and duration of residence have significantly increased the scientific impact of the paper. 

*** Reviewer #2:

A thorough set of responses, and helpful new analyses.

Some minor queries remain:

1. Can the authors provide some information in support of their statement that outpatient data was captured between 2001-2005? In other Swedish registry studies, it is stated that it is only captured after 2005.

**We have added the reference (35; Ludvigsson et al) to the sentence ”The National Patient Register begain recording in-patient psychiatric contacts in 1973 and outpatient contacts in 2001 [35]” .** **Lines 189-190, P8. We are unsure how else to demonstrate this, other than citation of several further studies which have used outpatient data since 2001, but this seems uncessary. For the reviewer’s benefit, see for example:** [**http://www.ncbi.nlm.nih.gov/pubmed/28270501**](http://www.ncbi.nlm.nih.gov/pubmed/28270501)**;** [**http://www.ncbi.nlm.nih.gov/pubmed/27086134**](http://www.ncbi.nlm.nih.gov/pubmed/27086134)**;** [**http://www.bmj.com/content/352/bmj.i1030**](http://www.bmj.com/content/352/bmj.i1030)**. Although earlier studies have stated that outpatient data were complete since 2001, recent information which has come to light suggests it is only safe to make this claim since 2006. We therefore erred on the side of caution here.**

2. The authors should add a comparison of the incidence reported in this study with other studies in high income countries. Without this, it is still not clear to what extent their incidence rates are underestimates (based on lack of outpatient and primary care data).

**We have addressed this by briefly commenting on the very limited incidence (vis-a-vis prevalence) data on this issue (lines 431-433, P20):**

**”Furthermore, these rates appear consistent with those observed in similar comprehensive national data in Denmark [14] and substantively higher than reported in general population samples in the Netherlands [41] and USA [42].”**

**References**

**41.	Bijl R V., de Graaf R, Ravelli A, Smit F, Vollebergh WAM. Gender and age-specific first incidence of DSM-III-R psychiatric disorders in the general population. Results from the Netherlands mental health survey and incidence study (NEMESIS). Soc Psychiatry Psychiatr Epidemiol. Steinkopff-Verlag; 2002;37: 372–379. doi:10.1007/s00127-002-0566-3**

**42.	Grant BF, Goldstein RB, Chou SP, Huang B, Stinson FS, Dawson DA, et al. Sociodemographic and psychopathologic predictors of first incidence of DSM-IV substance use, mood and anxiety disorders: Results from the Wave 2 National Epidemiologic Survey on Alcohol and Related Conditions. Mol Psychiatry. NIH Public Access; 2009;14: 1051–1066. doi:10.1038/mp.2008.41**

3. The new analyses find that those with PTSD are more likely to be diagnosed with substance use disorders. Can the authors expand on this? It is simply that those with PTSD are accessing healthcare and therefore are more likely to attract other diagnoses?

**Thank you. We have commented on this issue briefly on lines 483-486, P22 (additional text underlined):**

**”Finally, in line with another study, we found that people with a diagnosis of PTSD were more at risk of developing a substance use disorders [32], although further studies should investigate the extent to which these associations may result from greater healthcare contact and diagnostic assessment.”**

*** Reviewer #3:

Thanks authors for their efforts to improve the manuscript. I am satisfied with the response and the revision. No further issues needing attention.

***

**Production comments**

CC: [plosmedicine@plos.org](mailto:plosmedicine@plos.org)

Dear Dr. Kirkbride,

Thank you for submitting your revised manuscript (PMEDICINE-D-19-02064R1) entitled "Substance use disorders in refugee and migrant groups in Sweden: a nationwide cohort study of 1.2 million people" to PLOS Medicine.

Now that the Editor, Dr Philippa Berman , has informed you of the decision, the following production issues need to be addressed as well as any editorial requests before the manuscript can be formally accepted for publication.

Please address the following issues as soon as possible, remembering to include a marked up manuscript and a rebuttal letter addressing all editorial and production issues with your revision.

You may now have the option to publish your peer review history. If eligible, you will shortly receive an additional email with instructions on how to submit your choice in Editorial Manager. Please note that we are unable to register a choice on your behalf.

We look forward to receiving your manuscript by Sep 11 2019 11:59PM.

Production Issues:

FIGURES:

1. Please remove embedded figures from your manuscript file and ensure the most up to date versions of your figures are uploaded to our submission system as separate TIF or EPS files. Please do not remove the figure legends and captions.

**We have done so with Figure 1 (Fig1.tif).**

2. Please ensure that all main figure files are cited in ascending numerical order in the main text of the article.

**We have done so with Figure 1 (see “Fig 1. PRISMA flow diagram”, line 269, P11).**

3. Please ensure that all main figures are referenced in the manuscript as Fig 1, Fig 2, etc. (including capitalization), rather than Figure 1, Figure 2, etc. Please note, however, that the file names themselves must not include the space, i.e. Fig1.tif, Fig2.tif, etc.

**We have done so with Figure 1 (see “Fig 1. PRISMA flow diagram”, line 269, P11).**

4. Please ensure that all main figure legends are placed after the paragraph containing their first in-text citation.

**There is no legend associated with Figure 1.**

TABLES/BOXES:

1. Please cite main tables in ascending numerical order in your main article file.

**Tables are already cited in ascending numerical order in the text of the main article file.**

2. Please ensure that all main tables in their entirety (including titles and footnotes), are placed after the paragraph containing their first in-text citation.

**We have deleted all text “[Table X here]” (changes tracked) and moved the tables below the paragraph where they are first cited in the text (for clarity, moving the tables is not marked as a tracked change). Where tables overlap two pages, we have not corrected this.**

METADATA:

1. We still require confirmation of authorship and a declaration of any potential competing interests in relation to this manuscript from co-authors Anna-Clara Hollander and Christina Dalman. We have re-sent our authorship email, but also request that you contact these co-authors directly to ensure they respond.

**We have asked them to complete these forms.**

2. Please ensure that the CRediT author contributions listed for each co-author are completed in full. To qualify for authorship, each author must meet at least one of the seven core contributions (conceptualization; data curation; formal analysis; investigation; methodology; software; validation), as well as at least one of the writing contributions (original draft preparation; review and editing). Authors may also be noted against any of the remaining contributions, however these alone will not qualify a contributor for authorship.

Authors Anna-Clara Hollander are noted as contributing to "Funding acquisition" and "Writing – review & editing" only, which, as mentioned above, does not qualify them for authorship. Please ensure that the full contributions of each author are acknowledged in the "Add/Edit/Remove Authors" section of our submission form. If they do not meet criteria for authorship please let us know as soon as possible by emailing [plosmedicine@plos.org](mailto:plosmedicine@plos.org).

**We have updated their authorship contributions to correctly attribute their involvement and meet authorship guidelines. Prof Dalman was additionally involved in conceptualization of the study; Dr Hollander was additionally involved in data curation.**

3. Please ensure that the funders and grant numbers match between the Financial Disclosure field and the Funding Information tab in your submission form. Note that the funders must be provided in the same order in both places as well. Currently, you have listed all of your funders in both places, but they are listed in a different order.

**We have corrected the order in the Funding Information tab.**

4. In accordance with PLOS' data policy, please ensure that your Data Availability Statement in the submission form clearly identifies how readers can access your data. Data cannot be available on request, or only accessible by contacting one of the authors. Note that your Data Availability Statement will be typeset as it is written, so please ensure it is in complete sentences and appears as you would like it to in the published version.

For details on our data policy, please see <https://eur01.safelinks.protection.outlook.com/?url=http%3A%2F%2Fjournals.plos.org%2Fplosmedicine%2Fs%2Fdata-availability&amp;data=02%7C01%7C%7Cfaac4e4c98014ba1eb8c08d73557c483%7C1faf88fea9984c5b93c9210a11d9a5c2%7C0%7C0%7C637036523382465776&amp;sdata=UFcKsCxQpzveYdpXfbctkN4Zo%2FxahQ0%2FFf42YAmgfHQ%3D&amp;reserved=0>

**We have revised this statement in line with the editorial request above, and in compliance with Swedish law and ethical approval. See updated “Data availability” field in the submission form.**

ARTICLE BODY:

1. Please indicate the corresponding author in the byline by placing an asterisk (*) after their affiliation number.

**We have done so. See line 11, p1.**

2. Please include the corresponding author's email address on the title page of your manuscript, indicated by an asterisk (*). Only an asterisk and the email address itself are required.

**We have corrected this, line 24, p1.**

3. Please remove author degree titles from the byline of your article file.

**We have done so. Lines 10-11, p1.**

4. Please remove the funding information from your manuscript file. The published version is pulled automatically from the “Financial Disclosure” field in the submission form and must not be duplicated. Please therefore take this opportunity to check that the information in this field is correct and completed in full.

**We have done so.**

5. Please remove the Competing Interests section from your manuscript file. The published version is pulled automatically from the "Competing Interests" field in the submission form and must not be duplicated. Please therefore take this opportunity to check that the information in this field is correct and completed in full.

**We have done so.**

6. Please remove the data availability information from your manuscript file as this is automatically pulled from the "Data Availability" field in the submission form and must not be duplicated. Please therefore take this opportunity to check that the information in this field is correct and completed in full.

**We have done so.**

7. Please ensure that your Ethics statement is available in the Methods section of your manuscript in its entirety.

**We have added the ethics number to this statement (line 260, p10) and updated the Ethics Statement in the submission form to match this.**

ACKNOWLEDGMENTS:

1. Please remove the preprint link from the data statement and add it to the acknowledgements.

**We have added this to the (newly created) acknowledgements section (lines 546-548, p25).**

REFERENCES:

1. PLOS' policy regarding references is that only published or accepted manuscripts should be included in the reference list. Papers that have been submitted but not yet accepted should not be cited. Currently, your reference [44] is cited as follows: "Hollander A-C, Askegård K, Escalante C, Holmes EA, Wicks S, Dalman C. A validation study of cases of PTSD diagnoses identified in a Swedish regional database - Is the validity sufficient for epidemiological research? Under Review."

**We have removed this reference from the manuscript.**

SUPPORTING INFORMATION:

1. Please upload your supporting information as individual files, and use the following naming format: S1 Text; S1 Fig; S1 Table, S1 Data, etc. Each file type should start as S1 and increase numerically: S1 Text, S2 Text, etc. Note that all relevant in-text citations and captions will need to be updated accordingly.

**We have opted to use the approach above and submitted separate files.**

If you'd prefer to keep your supporting information in one file, please edit any citation of embedded figures and/or tables within your Supporting Information files and the main manuscript to use letters rather than numbers, e.g. “Table A in S1 Text” or "Fig B in S1 Appendix.".

2. Please ensure that all Supporting Information files use the following format verbatim throughout the manuscript (including legends and all in-text citations): S1 Fig, S1 Table, S1 Text, S1 Data, etc. Each file type should start at 1 and increase numerically. Please note, however, that the file names themselves must use an underscore rather than a space, i.e. S1_Fig.tif, S1_Table.xlsx, S1_Text.doc, S1_Data.xlsx, S1_STROBE_Checklist.docx, etc.

**We have renamed supplemental files accordingly, including “S1_Text.docx” (formally S1 Appendix) and “S1_STROBE_Checklist.docx” (formally S2 Appendix).**

3. Please ensure that each supporting information file has a legend at the end of the manuscript file, after the Reference list.

**We have added this. See P30.**

4. Please note that your submission cannot contain any references to generic "supporting information" files and instead must refer to specific files using our naming convention (S1 Fig, S1 Table, S1 Text, S1 Data, SI STROBE Checklist, etc.)

**We have renamed supplemental files accordingly, including “S1_Text.docx” (formally S1 Appendix) and “S1_STROBE_Checklist.docx” (formally S2 Appendix).**

-----

To edit your manuscript and the submission information provided with it you will need to login at <https://eur01.safelinks.protection.outlook.com/?url=https%3A%2F%2Fwww.editorialmanager.com%2Fpmedicine%2F&amp;data=02%7C01%7C%7Cfaac4e4c98014ba1eb8c08d73557c483%7C1faf88fea9984c5b93c9210a11d9a5c2%7C0%7C0%7C637036523382465776&amp;sdata=ZQEIigYpS8pHpbmRn9QUOg2kraZl8sptnKagU1AEJc0%3D&amp;reserved=0> and click the 'Submissions Needing Revision' link. In the list of action items click 'Edit Revision'.

Please note that once your paper is through to production it is difficult to alter the figures, so this is your best opportunity to ensure that they are correct for publication. It is also important to understand that any changes or additions to data are likely to require a further round of editorial review, which delays publication.

To make PLOS Medicine's open-access content as widely available as possible, we encourage authors to submit translated versions of their abstract (or, where desirable, the entire manuscript). If you do submit any translations when you resubmit your manuscript, they should be uploaded as Supporting Information files. Please don't hold up the resubmission of your paper just to obtain a translation: you can also provide it by email within a week of resubmission, but make sure you let us know who to credit for the translation.

If you have any questions, please feel free to get in touch.

Best regards,

Jessica Hyde

Publications Assistant

PLOS Medicine

[plosmedicine@plos.org](mailto:plosmedicine@plos.org)

__________________________________________________

In compliance with data protection regulations, you may request that we remove your personal registration details at any time. (Use the following URL: <https://eur01.safelinks.protection.outlook.com/?url=https%3A%2F%2Fwww.editorialmanager.com%2Fpmedicine%2Flogin.asp%3Fa%3Dr&amp;data=02%7C01%7C%7Cfaac4e4c98014ba1eb8c08d73557c483%7C1faf88fea9984c5b93c9210a11d9a5c2%7C0%7C0%7C637036523382465776&amp;sdata=DHfw3yECTopgHVtkgCl4WEQA8vn1acVZs%2BXH8Mg%2FuO8%3D&amp;reserved=0>). Please contact the publication office if you have any questions.
